# Links between fecal microbiota and the response to vaccination against influenza A virus in pigs

**DOI:** 10.1038/s41541-021-00351-2

**Published:** 2021-07-22

**Authors:** Marion Borey, Fany Blanc, Gaëtan Lemonnier, Jean-Jacques Leplat, Deborah Jardet, Marie-Noëlle Rossignol, Laure Ravon, Yvon Billon, Maria Bernard, Jordi Estellé, Claire Rogel-Gaillard

**Affiliations:** 1 Université Paris-Saclay, INRAE, AgroParisTech, GABI, Jouy-en-Josas, France; 2 INRAE, GenESI, Surgères, France

**Keywords:** Vaccines, Predictive markers, Inactivated vaccines, Biomarkers, Bacterial host response

## Abstract

This study describes the associations between fecal microbiota and vaccine response variability in pigs, using 98 piglets vaccinated against the influenza A virus at 28 days of age (D28) with a booster at D49. Immune response to the vaccine is measured at D49, D56, D63, and D146 by serum levels of IAV-specific IgG and assays of hemagglutination inhibition (HAI). Analysis of the pre-vaccination microbiota characterized by 16S rRNA gene sequencing of fecal DNA reveals a higher vaccine response in piglets with a richer microbiota, and shows that 23 operational taxonomic units (OTUs) are differentially abundant between high and low IAV-specific IgG producers at D63. A stronger immune response is linked with OTUs assigned to the genus *Prevotella* and family Muribaculaceae, and a weaker response is linked with OTUs assigned to the genera *Helicobacter* and *Escherichia-Shigella*. A set of 81 OTUs accurately predicts IAV-specific IgG and HAI titer levels at all time points, highlighting early and late associations between pre-vaccination fecal microbiota composition and immune response to the vaccine.

## Introduction

Worldwide, livestock production and animal welfare are threatened by a multitude of diseases. One of the main sanitary measures to defend animals against infections is vaccination, which aims to limit an individual’s susceptibility to infection and resulting infectiousness. By preventing disease at the level of the individual, vaccination subsequently protects herds and farms against widespread infections that can lead to high mortality and morbidity, and the potential of massive losses in production^1^. Thus, vaccination promotes animal welfare and helps to stabilize animal production costs. Furthermore, by preventing disease before it occurs, vaccination enables a reduction in antibiotic usage on farms, which mitigates the risk of promoting antibiotic-resistant bacteria^2^.

The swine flu is widespread around the world, affecting one out of every two farms in France^3^, and has a worldwide seroprevalence that ranges from 32% in Africa to 88% in South America^4^. The disease is usually benign but is highly contagious, with morbidity rates up to 100% on farms. Infected pigs have a fever and exhibit depression, coughing, sneezing, breathing difficulties, eye inflammation, and a lack of interest in feeding^5^. While mortality rates are generally low compared to other diseases (1–4%), swine flu leads to delayed weight gain, suboptimal reproductive performance, secondary infections, and thus economic losses. The etiologic agent is the Influenza A virus (IAV), a negative-sense single-stranded RNA virus belonging to family Orthomyxoviridae. The virus is characterized by its surface glycoproteins, hemagglutinin, and neuraminidase. Hemagglutinin enables the virus to enter into host cells, and neuraminidase leads to the release of virions from infected cells^6^. The main circulating strains of IAV are serotypes H1N1, H1N2, and H3N2^7,8^. In 2009, nucleic reassortment between the viral surface proteins of avian and porcine strains generated viruses that were also pathogenic to humans, leading to zoonosis and a pandemic^9^. Limiting outbreaks of swine flu is thus a global concern, with vaccination one of the best sanitary measures available^4^.

For most swine diseases for which a vaccine exists, there is variability in the response to vaccination within animal populations^10,11^. In order to increase the efficiency of vaccination, it is crucial to better understand the interactions between hosts and pathogens as well as the mechanisms of innate and adaptive immunity that are involved in the vaccine response. Likewise, it is important to investigate how these processes can vary among individuals, by deciphering the biological factors that explain why individuals within a single population differ in their ability to mount an immune response. Thus far, most genetic studies that have been conducted in pigs have shown that the host genetics influences the antibody response to vaccines against various pathogens^12–15^.

There is also growing evidence that the gut microbiota can play a role in the immune response to vaccines, at least in humans^16,17^, and these commensal and symbiotic microbial communities are currently the focus of research to improve vaccine design and efficiency^16–19^. The first advances in this field were obtained for oral vaccination and harnessed the adjuvant properties of specific types of intestinal bacteria. In humans and mice, it has been shown that the stability and survival of certain viruses in the gut are enhanced when the viral particles are attached to bacterial components. For instance, gut bacteria that express histo-blood group antigens stabilize norovirus in humans^20^, while bacterial N-acetylglucosamine and lipopolysaccharides (LPS) have a similar effect for poliovirus in mice^21,22^. Furthermore, the gut microbiota can have effects on remote parts of the body, and, for example, can act on the respiratory system to influence the disease severity of influenza or the vaccine response. Specifically, disequilibrium in the gut microbiota composition often referred to as dysbiosis, predisposes mice to severe influenza through changes in type I and II interferon and toll-like receptor signaling^23,24^; in humans, it alters the vaccine response of individuals with no pre-existing immunity to the flu virus^25^. Some of the underlying mechanisms have already been described. In the case of flu vaccination in humans, bacterial metabolites such as short-chain fatty acids^26^ or desaminotyrosine^27,28^, and specific components of the bacterial cell wall such as flagellin^29,30^, were shown to improve vaccine response. As a result of these findings, bacterial components are being increasingly considered as potential adjuvants to enhance the antigen persistence of inactivated vaccines^31^.

In pigs, our group has previously reported associations between pre-vaccination fecal microbiota and the response to vaccination against *Mycoplasma hyopneumoniae*. A small set of operational taxonomic units (OTUs), mainly assigned to genus *Prevotella*, was differentially abundant between individuals that demonstrated strong and weak responses to the vaccine^32,33^.

The main aims of our study were to analyse the following: (i) whether fecal microbiota composition before vaccination was associated with vaccine response intensity and (ii) whether vaccination induced differences in microbiota evolution between vaccinated and non-vaccinated pigs. To achieve these objectives, we investigated the links between the fecal microbiota and the individual humoral vaccine response of Large White pigs vaccinated against IAV. Animals were studied from just before vaccination until slaughter, and we followed the community dynamics of the fecal microbiota and the evolution of the immune response to the vaccine, measured by serum levels of IAV-specific IgG and assays of hemagglutination inhibition (HAI).

## Results

### An experimental design to follow both fecal microbiota composition and response to IAV vaccine over a period of four months

**Fig. 1:**
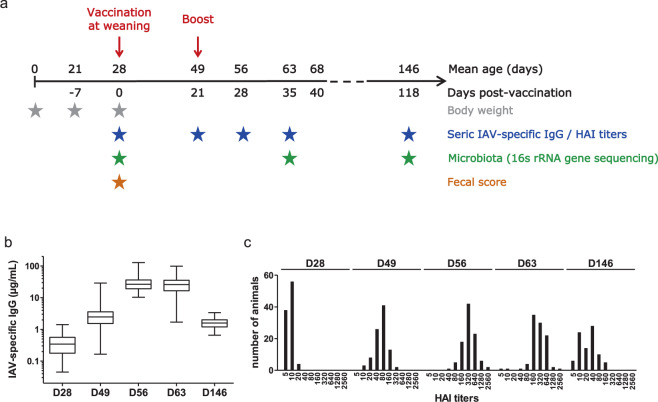
Experimental design and timeline of the Ab response after vaccination against IAV. **a** Summary of the protocol design, animal sampling, and data production. The timeline represents the mean age of the animals both from birth (above the line) and from the vaccine day (below the line). Animal parameters included body weight (gray stars) and fecal score at weaning on the vaccine day (red star). Stool samples were collected at three time points for 16S rRNA sequencing (green stars). In order to follow the vaccine response dynamics over time, blood was collected at five time points from the vaccine day until slaughtering (blue stars) for further seric dosage of IAV-specific IgG and measurement of HAI titers. **b** Box plot representation of IAV-specific IgG seric levels from vaccinated animals at D28 (before vaccination), D49, D56, D63, and D146. Box plots show minimum values, maximum values, median, and interquartile ranges. **c** Distribution of HAI titers measured from sera of vaccinated animals at D28 (before vaccination), D49, D56, D63, and D146.

We analyzed the association between fecal microbiota composition and vaccine response to IAV in a cohort of 98 pigs without passive immunization against IAV via the mother’s colostrum. The fecal microbiota was characterized by 16S rRNA gene sequencing and the vaccine response was assessed by measuring both seric levels of IAV-specific IgG and HAI titers (Fig. 1). The IAV-specific IgG levels follow the response to the overall influenza epitopes, and the HAI titers follow the response restricted to hemagglutinin that is the main target of neutralizing antibodies. As summarized in Fig. 1a, the pigs were vaccinated at 28 days of age (D28), with a booster three weeks later (D49). Stools and blood were collected at D28 before vaccination and at four time points corresponding to the early antibody response (D49), the maximum intensity antibody response (D56 and D63), and the persistence of antibody response until slaughtering (D146). Based on the range of variations for the vaccine responses measured by either seric IgG levels (Fig. 1b) or HAI titers (Fig. 1c), we identified at each time point groups of extreme responders (Table 1) that we defined as low and high responders (LR vs HR) as described in the Methods section. Due to the criteria chosen to set up the groups of high and low responders, the animals and their number per group vary according to time points and dosage. Despite a limited animal sharing across groups of extreme responders (Supplementary Fig. 1, Supplementary Data 1), we did not observe discordance for animals harboring high or low response at the three main periods of the response (early at D49, the maximum intensity at D56 or D63 and Ab persistence at D146). Then, depending on analyses, we studied either the whole population or groups of extreme responders based on IAV-specific IgG and HAI titers at D49, D56, D63, and D146.

**Table 1:** Average vaccine response at four time points post-vaccination.

Vaccine response phenotype	Age in days (dpv)	Total population		LR		HR	
		Mean or median titers^a^ [min-max]	*n*	Mean or median titers^a^ [min-max]	*n*	Mean or median titers^a^ [min-max]	*n*
IAV-specific IgG	D49 (21 dpv)	3248 [165–28,850]	98	702 [165–880]	9	14,704 [9135–28,850]	7
	D56 (28 dpv)	33,405 [10 300–128,500]	97	11,455 [10,300–12,300]	10	81,561 [60,050–128,500]	14
	D63 (35 dpv)	27,729 [4540–98,600]	95	9575 [4540–12,500]	11	55,070 [42,650–98,600]	12
	D146 (118 dpv)	1679 [651–3365]	89	902 [652–1050]	11	2768 [2310–3365]	14
HAI	D49 (21 dpv)	80 [10–320]	92	20 [10–20]	11	160 [160–320]	15
	D56 (28 dpv)	320 [40–2560]	97	160 [40–160]	24	640 [640–2560]	31
	D63 (35 dpv)	320 [40–2560]	95	160 [40–160]	40	640 [640–2560]	25
	D146 (118 dpv)	20 [5–160]	87	10 [5–10]	29	80 [80–160]	15

Data are provided as mean values for IAV-specific IgG (in pg/mL) and median values for haemagglutination inhibition assay (HAI) titers in the total population and in groups classified as high responders (HR) and low responders (LR).

*n* number of pigs in the total population and in each groups, *dpv* days post-vaccination.

^a^IAV specific IgG: mean; HAI: median titer.

### Variability of the pre-vaccination fecal microbiota in the vaccinated animals at D28

First, we analyzed the pre-vaccination (D28) fecal microbiota of 89 vaccinated piglets. Gene sequencing of 16S rRNA from fecal DNA provided 4,182,259 reads, which, following processing by FROGS, yielded 2,402,310 sequences. We obtained an average of 26,992 sequences per sample, with a range from 11,693 to 48,357. From these, we identified 1172 OTUs that were assigned to 10 phyla, 41 families, and 152 genera. An alignment of OTU sequences with the SILV132 16S rRNA sequence database provided annotation of the 10 phyla (100%), 37 of the 41 families (90.2%), 129 of the 152 genera (84.9%), and 40 out of 1172 OTUs (3.4%) (Supplementary Data 2). Eight OTUs — assigned to the families Ruminococcaceae, Prevotellaceae, Rikenellaceae, and Lachnospiraceae — were detected in all fecal samples. These may represent a core community at D28, and their cumulative average abundance reached 11.3% (Supplementary Data 2).

**Fig. 2:**
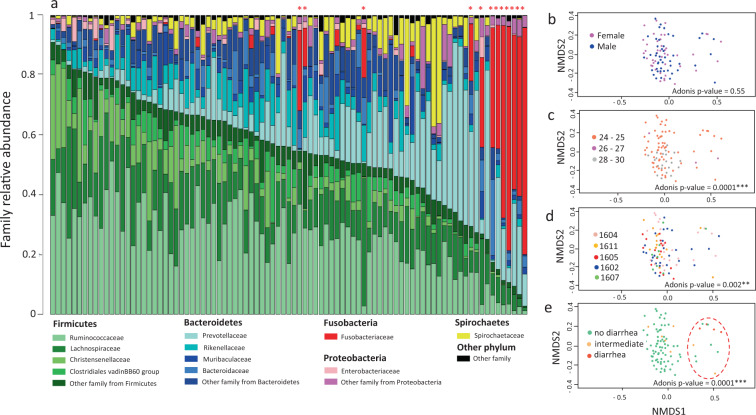
Composition of fecal microbiota of vaccinated piglets at D28 and visualization of sample dispersion by NMDS. **a** Relative abundance of OTUs in the fecal microbiota of 89 vaccinated pigs, annotated to the family level for families with a mean abundance over 2.5% and extended further for family Enterobacteriaceae. Each column represents a sample. Family annotation is color-coded and the corresponding phylum is indicated above the list of family names. The relative abundances of families are available in Supplementary Table 2. **b**–**e** NMDS representations of fecal microbiota sample dispersion considering the effect of **b** sex, **c** age at weaning in days, **d** batch, or **e** diarrhea score at D28. The corresponding *p*-values of the Adonis test are indicated. The 12 animals in which the relative abundance of Fusobacteriaceae was higher than 12% are surrounded with a red dotted circle on the NMDS representation in (**e**) and are highlighted by a red star on the abundance bar plot in (**a**). All corresponding data are available in Supplementary Table 1 and in Supplementary Data 3.

The two most dominant bacterial phyla were Firmicutes and Bacteroidetes, with average relative abundances of 54.8% and 30.5%, respectively; these were followed by Fusobacteria (6%), Spirochaetes (4.6%), and Proteobacteria (3.2%) (Supplementary Data 3). In phylum Firmicutes, the main families were Ruminococcaceae (25.5%), Lachnospiraceae (13.9%), and to a lesser extent, Christensenellaceae (6.2%) and Clostridiales vadinBB60 group (4.2%). In phylum Bacteroidetes, the main families were Prevotellaceae (12.4%), Rikenellaceae (6.2%), and to a lesser extent, Muribaculaceae (3.7%) and Bacteroidaceae (2.9%) (Fig. 2a).

As shown in Fig. 2b, there were no differences between the fecal microbiota of females and males at D28 (Adonis analysis on Bray Curtis distance, *p*-value = 0.55) (Supplementary Table 1). Body weight at weaning was the only parameter that displayed significant associations with alpha diversity. Animal batch, age at weaning, and weight at weaning were linked with dissimilarity in microbial community composition, as characterized with Bray Curtis distances (respective Adonis *p*-values: 1.6 × 10^−3^, 1 × 10^−4^, and 9.6 × 10^−3^) (Fig. 2c, d), with more homogeneity among the microbiota of animals that were older at weaning. The weaning age and body weight at weaning were not identified as confounding effects, as no significant correlation was observed between those two parameters (Pearson correlation coefficient = 0.15, *p*-value = 0.15). When we examined the community composition of the samples, we found that the 89 samples clustered in two unbalanced groups, which mainly differed in the relative abundance of Fusobacteria. Specifically, 12 samples had an abundance of Fusobacteria that was higher than 12%, while in the remaining 77 samples this phylum represented less than 0.55% of OTUs (Fig. 2e). Animals with a higher abundance of Fusobacteria displayed more diarrhea at D28 (Kruskall Wallis test, *p*-value = 6.8 × 10^−7^), but we did not have additional explanations for the presence of more Fusobacteria in this subset of 12 piglets.

### Fecal microbiota composition changes after weaning and vaccination, with no detectable differences on alpha and beta diversity between groups of vaccinated and non-vaccinated pigs

We compared the fecal microbiota of vaccinated (*n* = 89) versus non-vaccinated (*n* = 29) animals at all time points by analyzing patterns of alpha and beta diversity. Our results did not show significant effects of vaccination on alpha diversity (Richness and Shannon) or on beta diversity (Adonis analysis on Bray-Curtis distance) at any time point after vaccination (D49, D63, D146) (alpha diversity: all nominal *p*-values > 0.3, and FDR > 0.5; beta diversity: all nominal *p*-values > 0.2, and FDR > 0.9). Nevertheless, we were able to identify OTUs with differential abundance in vaccinated and non-vaccinated pigs at D28 (113 OTUs), D49 (74 OTUs), D63 (11 OTUs), and D146 (21 OTUs) (Supplementary Data 4). Almost all differentially abundant OTUs were rare (below 0.1%), except 2 OTUs out of 113 from D28 microbiota and 2 OTUs out of 74 from D49 microbiota that displayed a relative abundance between 1 and 2%. Because we detected such patterns of differential abundance between vaccinated and control animals even at D28 (Supplementary Data 4), it seemed that any differences between the two groups mostly arose from the wide range of inter-individual variability in fecal microbiota at weaning, and their overall effect was negligible. We were thus more focused on the differences in the pre-vaccination microbiota composition between high and low responders.

Of the original 98 vaccinated animals, we were able to obtain data at every time point for 74, meaning that we were able to follow the composition of their fecal microbiota from D28 to D146 (until slaughter). We identified 1258, 1207, and 1238 OTUs at D49, D63, and D146, respectively (Supplementary Data 2). Phylum Bacteroidetes was dominant at all ages post-weaning, with a mean relative abundance that declined with time from 64% to 48% (Supplementary Data 3). This group was mainly composed of Prevotellaceae. The next-most dominant phylum was Firmicutes, with a mean relative abundance that increased with time from 28 to 46%. This group was mainly composed of Lachnospiraceae, Ruminococcaceae, and to a lesser extent Christensenellaceae. Compared with the microbiota at D28, there was a large increase post-vaccination in the prevalence of the genus *Prevotella 9*, which reached a mean relative abundance of 44% at D63 and remained dominant until D146 (Supplementary Fig. 2). Of the other genera within family Prevotellaceae, five demonstrated more moderate increases after weaning (*Prevotella 7*, *Prevotella 2*, *Prevotellaceae NK3B31 group*, *Prevotella 1*, and an unknown genus), while *Dialister* and *Roseburia* also increased dramatically, from less than 0.6% to close to 5%. Conversely, *Christensenellaceae R-7 group*, *Fusobacterium*, *Ruminococcaceae UCG-005* and *002*, and *Bacteroides*, which were all among the ten most abundant genera in the D28 fecal microbiota, decreased to abundances of less than 5% after vaccination. We detected a stratification of the population into two enterotypes at D63 (Supplementary Fig. 3a-b, Supplementary Table 2), one with higher abundances of *Prevotella* and *Mitsuokella* (55 pigs) and the other with a higher abundance of *Treponema* (43 pigs).

### Alpha diversity of the pre-vaccination fecal microbiota at D28 is associated with the maximum intensity of the vaccine response at D56 and D63

Individual vaccine responses were evaluated at D49, D56, D63, and D146 by measuring IAV-specific IgG levels and HAI titers for the total population. Average responses are reported in Fig. 1b–c, and in Table 1. We then constructed a linear regression model between each alpha diversity index and the IAV-specific IgG and HAI responses from the total population of vaccinated animals. Based on this, we observed that fecal microbiota richness before vaccination displayed associations with vaccine response at D56 and D63 with a suggestive significance (nominal *p*-value < 0.05, FDR < 0.1). This was observed for both IAV-specific IgG and HAI: the higher the microbial richness, the higher the maximum vaccine response (Table 2, Supplementary Data 5).

**Table 2:** Association between the alpha and beta diversity of the D28 fecal microbiota and the IAV vaccine response at D49, D56, D63, and D146.

Vaccine response phenotype	Sampling day	Alpha diversity				Beta diversity	
		Richness		Shannon			
		*p*-value	FDR	*p*-value	FDR	*p*-value	FDR
IAV-specific IgG	D49	0.828	0.828	0.560	0.747	0.119	0.238
	D56	0.023*	**0.092**	0.051	0.373	0.322	0.429
	D63	0.036*	**0.096**	0.190	0.373	0.014*	0.112
	D146	0.436	0.581	0.656	0.750	0.194	0.310
HAI	D49	0.764	0.828	0.976	0.976	0.465	0.465
	D56	0.049*	**0.098**	0.197	0.373	0.079	0.238
	D63	0.016*	**0.092**	0.233	0.373	0.106	0.238
	D146	0.169	0.270	0.125	0.373	0.463	0.465

The vaccine response was assessed on the vaccinated population by seric IAV-specific IgG levels or haemagglutination inhibition assay titers (HAI). The whole population of vaccinated individuals was considered to estimate the association between the vaccine response and the alpha diversity indices. Association with beta diversity was assessed between the two groups of high and low responders to vaccine based on Adonis analysis on Bray-Curtis distances. Nominal *p*-values are reported in the table and highlighted in bold letters where significant (* for *p*-value < 0.05), FDR were calculated for each diversity metric independently and highlighted in bold letters were below 0.1.

### Groups of high and low producers of IAV-specific IgG at D63 differ by beta diversity and OTU content of their pre-vaccination fecal microbiota at D28

**Fig. 3:**
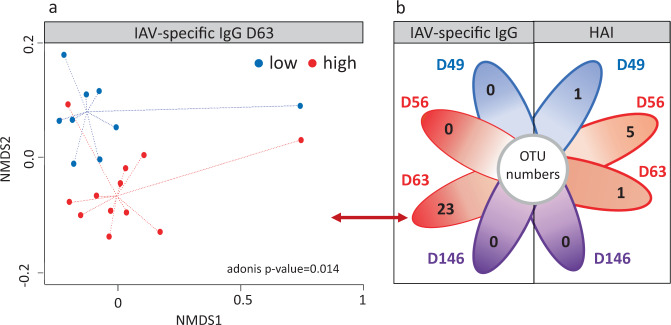
Links between the composition of pre-vaccination fecal microbiota at D28 and vaccine response levels at different time points. **a** NMDS representation of individuals corresponding to high (red dots) and low (blue dots) producers of IAV-specific IgG at D63. **b** Number of OTUs that were differentially abundant between high and low responders defined by IAV-specific IgG or HAI at D49 (blue), D56 (red), D63 (red), and D146 (purple). Arrows connect the number of differentially abundant OTUs with the NMDS representation of the corresponding high and low groups used for the differential abundance analysis. NMDS ordination is based on Bray-Curtis distance. The *p*-values of the Adonis test between high and low groups are indicated (corresponding data available in Table 2 and Supplementary Data 6).

Based on their IAV-specific IgG levels or HAI titers at D49, D56, D63, and D146, pigs were classified as high vaccine responders (HR) or low vaccine responders (LR). The average vaccine responses for those groups are reported in Table 1. We then investigated potential links with their microbiota by calculating the microbial dissimilarity at D28 between HR and LR pigs (Table 2). The only significant difference in community composition was found between HR and LR groups defined by IAV-specific IgG levels at D63, as revealed by the presence of two clusters in an NMDS ordination (Fig. 3a, Adonis *p*-value = 0.014). Of the two outliers, one represented HR and the other LR, and both corresponded to piglets harboring a high abundance of Fusobacteria. Although we detected five differentially abundant OTUs between HR and LR groups defined by HAI response at D56, there was only a trend toward dissimilarity between these groups based on Bray-Curtis distances (*p*-value = 0.079).

We carried out an analysis of D28 fecal microbiota to identify OTUs that were differentially abundant between the groups that would later demonstrate extreme responses. As shown in Fig. 3b, we found 23 differentially abundant OTUs between the HR and LR groups defined by IAV-specific IgG at D63, 5 differentially abundant OTUs between the groups defined by HAI at D56, and 1 differentially abundant OTU for the groups defined by HAI at D49 (Supplementary Data 6). No differentially abundant OTUs were observed at any other time point.

**Fig. 4:**
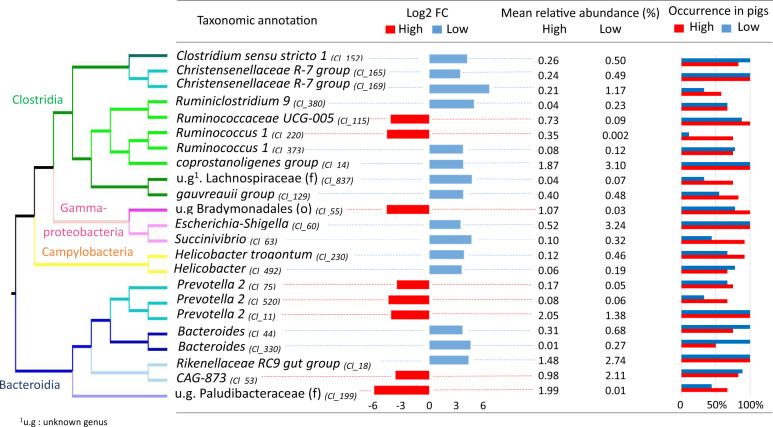
Summary of taxonomic annotation and quantitative analysis of the 23 OTUs that were differentially abundant in the D28 fecal microbiota of pigs that demonstrated extreme IAV-specific IgG responses at D63. OTUs are ordered according to their phylogeny. Taxonomic classification is indicated at the genus level except for two OTUs annotated at the levels of family (f) and order (o). The cluster identifier for each OTU is indicated in parentheses after the taxonomic annotation. The Log2 fold change (Log2 FC) between high (red) and low (blue) responders is represented by horizontal histograms together with the mean relative abundance, in percentage (%), per group. The respective occurrence of each OTU in high (red) and low (blue) responder groups is indicated on the right hand of the figure and indicates the percentage of individuals per group that contained that OTU. All data are available in Supplementary Data 6.

When we examined the set of 23 OTUs that were differentially abundant in the D28 microbiota of extreme responders for IAV-specific IgG at D63, 8 were more abundant in the future HR group and 15 were more abundant in the future LR group (Fig. 4). Among the eight OTUs that were overabundant in the HR group, three corresponded to unknown species assigned to the genus *Prevotella 2*: Cluster_520 (FDR 0.04), Cluster_11 (FDR 0.004), and Cluster_75 (FDR 0.03) (Supplementary Fig. 4a). Cluster_11 was present in all piglets, with average abundances of 2.05% and 1.38% in HR and LR groups, respectively. An unknown species from family Paludibacteraceae had the most significant and largest-magnitude change between the two groups of extreme responders, with a mean abundance of 2% among HR animals and 0.01% among LR animals (Cluster_199, FDR = 0.0008, Log2FC = −5.07). An OTU assigned to order Bradymonadales was found with an average abundance of 1.07% in the HR group and 0.03% in the LR group (Cluster_55, FDR = 0.004). Three other OTUs from the genera *Ruminococcaceae UCG-005*, *Ruminococcus 1*, and *CAG-873* were specific to the HR animals, but with mean relative abundances below 1% (Cluster_220, Cluster_115, and Cluster_53, respectively).

Among the 15 OTUs that were more abundant in the LR group, two were annotated to genus *Helicobacter* (Cluster_230 FDR = 0.02, Cluster_492 FDR = 0.03), two to genus *Bacteroides* (Cluster_44 FDR = 0.03, Cluster_330 FDR = 0.02), two to *Christensenellaceae R-7 group* (Cluster_169 FDR = 0.004, Cluster_165 FDR = 0.03), one to *Succinivibrio*, and one to *Escherichia-Shigella*. The mean relative abundance of the OTU assigned to *Escherichia-Shigella* reached 3.3% in the LR group, and no other OTU from this genus was identified in the dataset (Cluster_60, FDR = 0.03) (Supplementary Fig. 4b). This OTU was found in all piglets (HR and LR groups), meaning that the difference between the two groups was due to differential abundance, and was not biased by the absence of this OTU in the HR group.

**Fig. 5:**
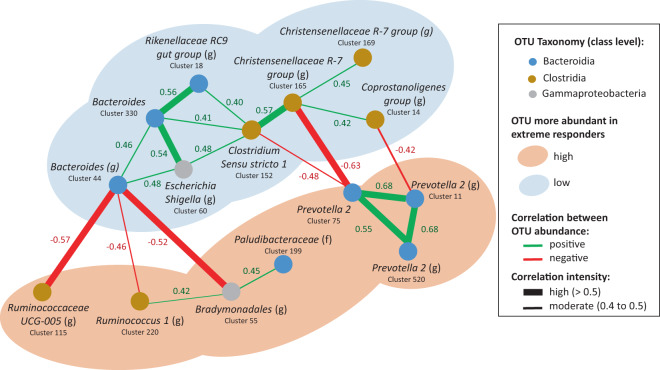
Co-abundance network of OTUs that were differentially abundant in the D28 fecal microbiota of pigs that demonstrated extreme IAV-specific IgG responses at D63. Only intermediate (fastsparr correlation > 0.4) or high (fastsparr correlation > 0.5) correlations of statistically significant effect (*p*-value < 0.05) are represented, reducing the initial number of 23 differentially abundant OTUs to 15 in the figure. OTUs that were more abundant in high or low responders are surrounded by pale orange or pale blue ovoids, respectively. Each OTU is represented by a node with a color that corresponds to its class affiliation (blue for Bacteroidia, brown for Clostridia, and gray for Gammaproteobacteria). Each OTU is named with its corresponding sequence cluster identifier with annotation at the genus (g) or family (f) level. Co-abundance between two OTUs is represented by a connecting line, green or red for positive and negative correlations, respectively. The thickness of the connecting lines is proportional to the strength of the correlation, either between 0.4 and 0.5 or above 0.5. Correlation was calculated on the whole population of 89 vaccinated piglets and their associated p-values are available in Supplementary Data 7.

A network analysis with the 23 differentially abundant OTUs (Fig. 5) allowed us to evaluate if there were groups of co-abundant OTUs that could act together or share similar ecological niches. For the OTUs that were more abundant in the HR animals, we observed that the relative abundances of the three OTUs annotated to *Prevotella 2* were highly correlated (*p*-value = 0.02, rho estimator Cluster_75-Cluster_520 = 0.55, Cluster_75-Cluster_11 = 0.68, Cluster_520-Cluster_11 = 0.68) (Supplementary Data 7). Cluster_55, which was assigned to order Bradymonodales, and Cluster_199, assigned to family Paludibacteraceae, were also co-abundant (*p*-value = 0.02, rho estimator = 0.45). For those that were more abundant in the LR group, at the genus level, we observed co-abundance between two OTUs annotated to the *Christensenellaceae R-7 group*, and between two OTUs annotated to genus *Bacteroides*. When we investigated associations among OTUs with close phylogenetic relationships, we observed that an OTU from *Christensenellaceae R-7 group* (Cluster_165) was co-abundant with three other Clostridia (Cluster_169, 14, and 152), and that three OTUs from genus *Prevotella 2* were all co-abundant, with high correlation coefficients (Cluster_75, 11, and 520).

### Identification of a set of 81 OTUs from D28 fecal microbiota that predicts the vaccination response level

We applied sPLS-DA discriminant analyses to study whether OTUs identified in the D28 fecal microbiota could serve as predictors of the LR and HR groups defined by IAV-specific IgG and HAI at D49, D56, D63, and D146 (Supplementary Data 8).

**Fig. 6:**
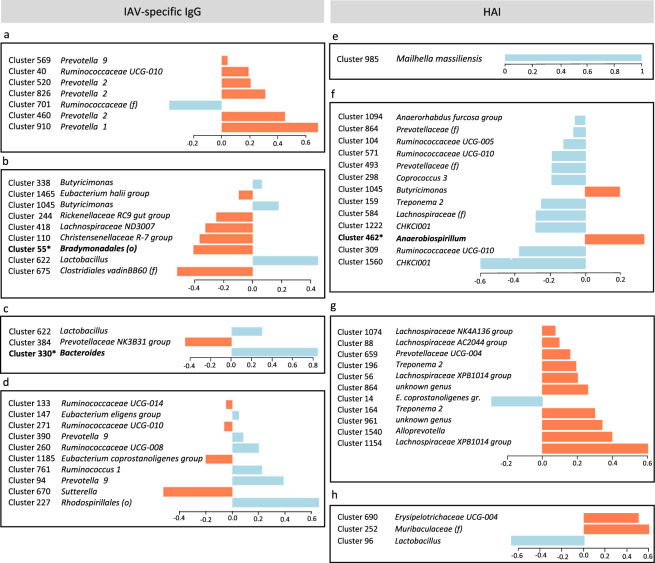
Loading plots representing the specific contribution of OTUs to the prediction of vaccine response. The OTUs were selected by sPLS-DA (data available in Supplementary Data 8). The color of each bar indicates the group of pigs in which that OTU had a higher mean relative abundance: orange for high responders and blue for low responders. **a**–**d** Contributions of OTUs to the first component are displayed for the analysis of IAV-specific IgG levels at **a** D49, **b** D56, **c** D63, and **d** D146. **e**–**h** Contributions of OTUs to the first component are displayed for the analysis of HAI titers at **e** D49, **f** D56, **g** D63, and **h** D146. OTU annotation is indicated at the genus level and, where not available, at the family (f) or order (o) levels. Asterisks indicate the few OTU that were differentially abundant between high and low responders at the same time points post-vaccination.

We identified sets of 7, 9, 3, and 23 OTUs that best predicted high or low levels of IAV-specific IgG at D49 (AUC = 0.952, *p*-value of 0.007), D56 (AUC = 0.959, *p*-value of 8 × 10^−4^), D63 (AUC = 0.982, *p*-value of 2 × 10^−4^), and D146 (AUC = 1, *p*-value of 6 × 10^−5^), respectively (Fig. 6a–d, Supplementary Data 8). Among the seven OTUs that best-discriminated vaccine response at D49, five were annotated to family Prevotellaceae, of which three were assigned to the genus *Prevotella 2* and the remaining two to the genera *Prevotella 1* and *9*. This was consistent with our finding that OTUs assigned to *Prevotella 2* were found to be differentially abundant in the HR group defined by IAV-specific IgG at D63 (Fig. 4). One OTU annotated to family Prevotellaceae was also identified among the three OTUs that best discriminated among the extreme responders at D63. Two OTUs assigned to *Prevotella 9* were also found to be highly predictive of IAV-specific IgG response at D146. However, 10 of the 23 most-discriminant OTUs were assigned to family Ruminococcaceae. One OTU (Cluster_622) from genus *Bacteroides* was also identified through sPLS-DA among the nine bacteria that distinguished the extreme IAV-specific IgG responders at D56.

By sPLS-DA analysis, we also identified sets of 5, 16, 22, and 3 OTUs that best predicted high or low levels of HAI at D49 (AUC = 1, *p*-value of 1 × 10^−4^), D56 (AUC = 0.923, *p*-value of 4 × 10^−7^), D63 (AUC = 0.953, *p*-value of 5 × 10^−9^), and D146 (AUC = 0.821, *p*-value of 7 × 10^−4^) (Fig. 6e-h, Supplementary Data 8). In predicting the response at D49, the two strongest contributors were OTUs from the genera *Mailhella* and *Lactobacillus*. At D56 and D63, the five most-predictive taxa were the families Lachnospiraceae (4 and 5 OTUs) and Ruminococcaceae (4 and 2 OTUs), followed by Muribaculaceae (2 and 3 OTUs), Prevotellaceae (2 and 5 OTUs), and Spirochaetaceae (1 and 4 OTUs). All OTUs annotated to family Spirochaetaceae were assigned to the genus *Treponema 2*. Interestingly, the OTU defined by Cluster_53 (Muribaculaceae), which contributed to the prediction of the response at both D56 and D63, was also present in the set of five OTUs that were differentially abundant between HR and LR groups at D56. For prediction of the response at D146, the three OTUs highlighted were annotated to the families Lactobacillaceae, Muribaculaceae, and Erysipelotrichaceae.

Three OTUs were found to be predictive of both IAV-specific IgG and HAI. Cluster_1094, annotated to family Erysipelotrichaceae, was predictive of HAI response at D56 and IAV-specific IgG response at D63. Cluster_1045, annotated to genus *Butyricimonas*, was predictive of HAI response at D56 and IAV-specific IgG response at D56, and Cluster_418, from family Lachnospiraceae, was predictive of HAI response at D63 and IAV-specific IgG response at D56.

**Fig. 7:**
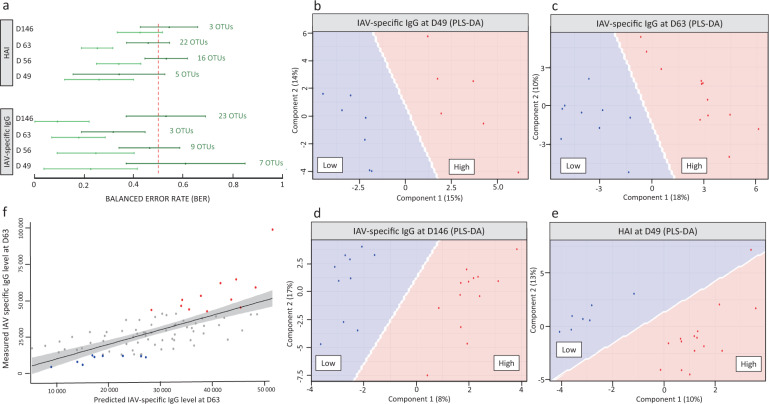
Predictive capacity of OTUs in pre-vaccination D28 fecal microbiota for vaccine response measured by IAV-specific IgG and HAI. **a** Comparison of the prediction yielded by the OTU subsets found at each time point for each vaccine response phenotype (sPLS-DA, dark green line, indication of the number of OTUs selected for each prediction) and by the combined set of 81 OTUs (PLS-DA, light green line). The BER estimations are represented by their 95% confidence intervals, with their means ± 2 standard deviations. **b**–**e** Projection of the groups of extreme responders to the influenza vaccine as measured by IAV-specific IgG at **b** D49, **c** D63, **d** D146, and **e** HAI titers at D49, obtained with a PLS-DA implemented with the combined set of 81 candidate predictive OTUs. Samples are classified as low responders (blue dots) or high responders (red dots). Corresponding sPLS-DA and PLS-DA data are available in Supplementary Table 4 and in Supplementary Data 1. **f** Plot of measured vs. predicted IAV-specific IgG titers at D63 from a regression model. Predicted values were estimated with PLS analysis based on the counts of the combined set of 81 OTUs applied to the population of all vaccinated piglets that had both a characterized fecal microbiota at D28 and a measured titer of IAV-specific IgG at D63 (*n* = 86). Low vs. high responders for IAV-specific IgG at D63 are represented with blue and red dots, respectively; all other animals are represented by gray dots.

We gathered all predictive OTUs that were highlighted by sPLS-DA for the four response time points to create a list of 81 unique OTUs. With these, we performed a PLS-DA analysis between the LR and HR groups for IAV-specific IgG and HAI at D49, D56, D63, and D146 (Supplementary Data 9). In comparison with the initial sPLS-DA, this analysis improved the predictions of the two types of the immune response at each time point (Fig. 7a). The best prediction scores were obtained, at all time points, for IAV-specific IgG levels compared to HAI titers. Specifically, the highest prediction scores were observed for IAV-specific IgG levels at D146 (AUC = 1, BER + 2 SD = 0.22) (Fig. 7d) and at D63 (AUC = 1, BER + 2 SD = 0.29) (Fig. 7c), and to a lesser extent for IAV-specific IgG levels at D49 (AUC = 1, BER + 2 SD = 0.41) (Fig. 7b) and HAI at D49 (AUC = 1, BER + 2 SD = 0.40) (Fig. 7e).

Finally, by applying a PLS to the entire population (*n* = 89) using this set of 81 predictive OTUs (Fig. 7f), we were able to predict the IAV-specific IgG response at D63; the observed Q2 value (goodness of prediction) was 0.095, below the recommended limit of 0.0975. Furthermore, we observed a strong correlation between the predicted and measured values (Pearson correlation = 0.70, *p*-value = 6.5 × 10^−14^), and verified the normality of their residuals through a Shapiro-Wilcoxon test (*p*-value > 0.05).

### No detection of an OTU signature specific of high and low vaccine responses maintained overtime

**Fig. 8:**
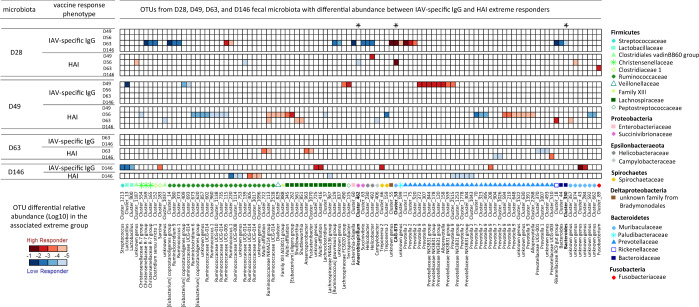
Representation of the 102 OTUs identified as differentially abundant in the fecal microbiota of 28, 49, 63, and 146-day-old pigs between groups of high and low responders for IAV-specific IgG and HAI at all time points post-vaccination. The mean OTU relative abundance per group is represented with a color gradient. OTUs that were more abundant within the high responder groups are represented with a red gradient, and OTUs that were more abundant within low responder groups are represented with a blue gradient. Asterisks highlight the three OTUs that were also identified by sPLS-DA as predictors of vaccine response at the same time point for the same vaccine phenotype. Taxonomic annotation is given at the genus level. All corresponding data are available in Supplementary Table 3 and in Supplementary Data 6.

We analyzed whether the fecal microbiota at D49, D63, and D146 also contained OTUs that were differentially abundant between extreme vaccine responders at D49, D56, D63, and D146 (Supplementary Table 3). We found no association between the enterotypes at D63 and extreme vaccine response at D63 or D146 (chi test, *p*-value < 0.05). Figure 8 provides a global overview of the entire set of 102 OTUs that were differentially abundant, at any time point, in the fecal microbiota of HR and LR groups defined by IAV-specific IgG or HAI. The figure also includes the OTUs from the D28 microbiota that were identified by sPLS as candidate predictors of vaccine response levels. From D49 fecal microbiota data, there were 13 differentially abundant OTUs between the extreme responders defined by IAV-specific IgG at D49, and 28 and 7 OTUs differentially abundant between extreme responders defined by HAI at D56 and D63, respectively. From D63 fecal microbiota data, we did not detect any OTUs that were differentially abundant between HR and LR groups defined by IAV-specific IgG at D63 and D146. However, when HR and LR groups were defined by HAI levels, we found 7 and 3 differentially abundant OTUs at D63 and D146, respectively. Finally, from D146 fecal microbiota data, we identified 10 and 11 differentially abundant OTUs between HR and LR groups defined by IAV-specific IgG levels and HAI titers, respectively. Most of these OTUs had relative abundances below 0.1%, which stood in contrast with the differentially abundant OTUs from the D28 fecal microbiota of extreme responders for IAV-specific IgG at D63.

There was no overlap between OTUs that were differentially abundant at D28 and at later time points; we observed two OTUs that remained present in later microbial assemblages, but the later patterns of abundance contrasted with those detected at D28 (Cluster_115, Cluster_18). For time points later than D28, seven OTUs were shared between two analyses, and a single OTU (Cluster_113, Ruminococcaceae family) was identified in three analyses: it was overabundant in the D49 microbiota of HR for HAI at D56 and D63, and in the D63 microbiota of HR for HAI at D63. Finally, from the list of 81 predictors of vaccine response based on their abundance in the D28 microbiota, three OTUs (Cluster_462 from genus *Anaerobiospirillum*, Cluster_53 from family Muribaculaceae, and Cluster_330 from genus *Bacteroides*) were also differentially abundant in the D28 fecal microbiota between HR and LR groups for IAV-specific IgG level at D63 or HAI titer at D56.

## Discussion

The efficiency of vaccination is a major concern in efforts to ensure individual- and herd-level protection against specific pathogens. If too many individuals produce low vaccine responses with limited protection, herd immunity may not be achieved, leading to disease outbreaks. Research on vaccine response has implicated many factors, including host genetics, age, and the environment^34^. Here, we explored the role of the gut microbiota, which in humans and mice has been shown to influence immune responses to vaccination and the inter-individual variability thereof^16,35^. We confirmed a relationship between the pre-vaccination gut microbiota in young pigs and their later immune responses to the IAV vaccine; overall, these findings were similar to those of our previous work on piglets vaccinated against *Mycoplasma hyopneumoniae*^32,33^. Specifically, the current study observed links between the pre-vaccination fecal microbiota of 28-day-old piglets and their response to vaccination against IAV, as assessed by serum levels of anti-IAV IgG and HAI. Our main findings highlighted the beneficial association of OTUs assigned to genus *Prevotella* and family Muribaculaceae with a stronger individual vaccine response, and allowed us to identify candidate OTUs to predict the strength of that response.

In addition to the analysis of pre-vaccination fecal microbiota, we also investigated possible links between vaccination and fecal microbiota dynamics from D28 until slaughter at D146, and followed the evolution of the fecal microbiota in individuals that demonstrated extreme responses to IAV vaccination. From D49 to D146, we did not observe any significant associations between the alpha or beta diversity of the fecal microbial assemblages and the level of vaccine responses based on the analysis of HR and LR groups. We did, however, identify OTUs in the fecal microbiota at D49, D63, and D146 that were differentially abundant between the two extremes of vaccine response, but almost all these OTUs differed according to each time point. These data did not allow us to characterize a microbial signature that might follow a high or low response to vaccination over life. We did not either observe any association between enterotypes at D63 and vaccine response (HR versus LR) at D63 or D146, despite previous reports that enterotypes at 70 days of age are associated with levels of luminal secretory IgA^36^. In our study, the vaccine was first injected at D28 on the weaning day at a time when the gut microbiota is shifting due to the transition from milk feeding to a solid cereal-based diet. As already shown in Large White pigs bred in the same environment^32,36^, our data set confirmed such a shift before and after weaning. Nevertheless, the set of 81 predictive OTUs identified in the D28 fecal microbiota provided consistency over time for associations between OTU abundances at D28 and further vaccine responses.

A comparison of the vaccinated and non-vaccinated groups showed that 74, 11, and 21 OTUs were differentially abundant in the microbiota at D49, D63, and D146, respectively. However, their relative rarity provides evidence that intramuscular vaccination against IAV at D28 did not modify the overall dynamics of the microbiota in the long term. Based on this, we conclude that vaccination may have a slight effect on the fecal microbiota, but one that is mainly visible early after vaccination and barely detectable at slaughter.

Interestingly, our results revealed associations between the pre-vaccination fecal microbiota at D28 and the maximum intensity of the vaccine immune response measured at D56 and D63, two and three weeks after the vaccine booster, respectively. Specifically, we found that the higher the fecal microbiota richness, the higher the levels of IAV-specific IgG and HAI at D56 and D63. These results thus extend to pigs a hypothesis that has been proposed for various species: higher microbial richness and diversity, which are often linked to eubiosis and host homeostasis, favor a more efficient immune response to vaccination^17^. For example, in a clinical study of human adults vaccinated against *Salmonella enterica* serovar Typhi, a more diverse microbial community was observed in individuals who displayed an early and persistent cell-mediated immune response to the vaccine, while individuals with less diverse microbiota instead displayed a late cell-mediated immune response^37^. Similarly, macaques whose gut microbiota were characterized by higher Shannon diversity also demonstrated higher vaccine effectiveness against *Shigella dysenteriae*^38^, and infant mice with artificially depressed intestinal bacterial diversity subsequently displayed a lower IgG response to several vaccines directed against diverse bacterial pathogens^39^. A richer and more diverse microbiota has been frequently linked with health benefits for hosts, and many authors have explained this effect as an enhanced capacity for resilience. Increased resilience means that minor or even moderate stresses are less likely to result in a shift of the bacterial community, and in case of perturbation, these communities return to their initial basal composition more quickly^40^. A higher capacity for resilience may also have beneficial effects on immunological efficiency. Thus, optimization of gut microbiota richness could be an interesting tactic for strengthening the overall immune system and the vaccine response in particular.

We found a significant association between the beta diversity of fecal microbiota at D28 and serum levels of IAV-specific IgG at D63; this relationship was further reinforced by the identification of 23 OTUs that were differentially abundant between HR and LR groups defined by IAV-specific IgG at this time point. Among the eight OTUs that were more abundant in the HR group, three were assigned to the genus *Prevotella 2*, which suggests a role for this bacterium in the improvement of the immune response to the IAV vaccine. Similarly, among a set of seven OTUs identified as good predictors of high levels of IAV-specific IgG at D49 (sPLS-DA, component 1), three were assigned to *Prevotella 2*, one to *Prevotella 1*, and one to *Prevotella 9*. Interestingly, though, among the 20 OTUs identified as good predictors of low levels of IAV-specific IgG at D146 (sPLS-DA, components 1 and 2), two were also assigned to *Prevotella 9*. This pattern of results — on the one hand, indication of a beneficial role for *Prevotella* but on the other, show that a few OTUs assigned to the same genus might have the opposite associations — is similar to previously published data from a study of immune responses to vaccination against *M. hyopneumoniae*^32,33^. In the work of Munyaka et al.^32^, the piglets vaccinated against *M. hyopneumoniae* were from the same families as in the present study, and were vaccinated simultaneously. In these two vaccination studies, all piglets were Large White, shared the same environment, and were fed a similar diet. By contrast, in the earlier study of Munyaka et al.^33^, the animals were (Large White x Landrace) x Duroc piglets that were bred and vaccinated against *M. hyopneumoniae* in Canada, and thus differed from the two latter studies in animal genetics, country, and the rearing environment. Despite these differences, however, the data from this earlier work also revealed links between a higher abundance of OTUs assigned to *Prevotella* and a higher immune response to the *M. hyopneumoniae* vaccine. Notably, sequence alignment revealed that the OTUs identified in Munyaka et al.^32^ were likely distinct from the OTUs identified in this study despite being assigned to the same genus. Taken together, all of these results suggest a generally beneficial role of bacteria from genus *Prevotella* for the vaccine response to two different pathogens (IAV and *M. hyopneumoniae*). However, it will be necessary to characterize these bacteria in pigs at the level of the species or even the strains in order to enable fine-scale discrimination between more- and less-favorable species/strains from the same genus. This becomes all the more important given our observation of up to 312 OTUs annotated to a *Prevotella*-type genus in pig fecal microbiota, and our finding of a stark contrast between the relative abundances of OTUs assigned to the genera *Prevotella 2* and *Prevotella 9*. The relative abundance of *Prevotella 2* increased slightly after weaning, but remained more stable over pigs’ lifespans than that of *Prevotella 9*, which became by far the most abundant genus until slaughter. There are already many reports that implicate *Prevotella* bacteria in the vaccine response in other species. In humans, nasal abundance of *Prevotella melaninogenica* was found to correlate with influenza antibody titers^41^, while another study reported that genus *Prevotella* drives the Th17 immune responses through the activation of Toll-like receptor 2^42^. In mice vaccinated against the hepatitis B virus, bacteria in the genera *Bacteroides* and *Prevotella* generated lipopolysaccharides (LPSs) that demonstrated adjuvant properties^43^. In pigs, it is already known that *Prevotella* bacteria produce butyrate, a short-chain fatty acid that favors the development of the immune system, plasma cell differentiation, and antibody response^44^. Thus, although the underlying functionalities remain to be elucidated in pigs, it seems possible that bacteria belonging to genus *Prevotella* might also have a role in modulating innate immunity.

The OTU Cluster_53, which was annotated to the genus *CAG 873* within family Muribaculaceae, was also of interest for its potential benefits. This OTU was significantly more abundant in the D28 fecal microbiota of piglets that had high levels of IAV-specific IgG at D63 and HAI at D56, and was in the list of OTUs that best predicted a strong HAI response at D56. As a result of constant improvement in bacterial annotation in the 16S rRNA reference sequence databases, family Muribaculaceae was recently renamed; depending on the database and report in question, OTUs in this family had previously been assigned to one of four other families: ‘S24-7’ in release 128 of the SILVA database, ‘Porphyromonadaceae’ in the RDP release, and ‘MIB’ (mouse intestinal bacteria) or Homeothermaceae in the literature^45^. Interestingly, bacteria from the S24-7 family were also reported to be more abundant in piglets with a stronger vaccine response to *Mycoplasma hyopneumoniae*^32^. This family is similarly associated with good health in mice, and was reported to be significantly depleted in individuals with colitis^46^. Previous predictions using PICRUSt, which infers the functional metagenome of a bacterial community based on its taxonomic composition as assessed through 16S rRNA gene sequencing, have suggested that the S24-7 family contains peptidases known for their immunomodulatory activity^47,48^. Like *Prevotella*, S24-7 bacteria are Gram-negative (LPS is a main component of the bacterial wall) and produce butyrate. Thus, it would be interesting to further explore their possible role as promoters of good health, and even consider them as new probiotic candidates in pigs, especially since bacteria from the Muribaculaceae have recently been successfully cultured^45^.

Among the 15 OTUs that were more abundant in the D28 fecal microbiota of pigs with weak IAV-specific IgG responses at D63, we identified the genera *Helicobacter*, *Bacteroides*, and *Christenellaceae R7 group*. Two OTUs corresponded to the species *Helicobacter trogontum*, while seven others were annotated to the genera *Escherichia-Shigella*, *Succinivibrio*, *Clostridium sensu stricto 1*, *Ruminiclostridium 9*, *Ruminococcus 1*, *coprostanoligenes group, gauvreauii group, or Rikenellaceae RC9 gut group*. Many bacteria from this list are known to cause pathogenic disorders, and have been associated either with clinical illness or with a perturbation of the immune system. *Helicobacter trogontum* can cause colitis in immune-compromised patients^49^, invade human enterocytes, and induce hepatic inflammation in mice^50^. Genus *Helicobacter* also contains other pathogenic bacteria, and its worldwide prevalence in the stomachs of pigs at the age of slaughter has been reported to be, on average, higher than 60%^51^. Pigs are commonly infected with the species *Helicobacter suis or Helicobacter pylori*^52^, which both lead to gastric inflammation and decrease daily weight gain^53^. Although the animals in the present study did not show any clinical signs of infection, our results suggest that the two OTUs annotated to *Helicobacter* might have induced low levels of inflammation in the digestive tract and thus impaired the strength of the immune response to vaccination. With respect to *Bacteroides*, another predictor of weak IAV-specific IgG response at D63, our results were consistent with evidence from the literature demonstrating a role for these bacteria in immune inhibition. Bacteria from this genus were more abundant in the fecal microbiota of babies that displayed a weak response to Rotavirus vaccination^54^, and this taxon is the main culprit behind bacteraemia and systemic blood infections in adult patients^55^. Since *Bacteroides* have less immunogenic LPS than other Gram-negative bacteria, it has been suggested that a higher abundance of this genus could lead to reduced stimulation of the innate immune system^56^. Thus, we can hypothesize that the two OTUs from genus *Bacteroides* that were identified in our study may have hampered the maturation of the immune system, with consequences for vaccine efficacy. An OTU from *Clostridium sensu stricto 1* was also present in the list of best predictors of a weak IAV-specific IgG response at D146; in a previous study, the relative abundance of this genus was positively correlated with diarrhea and negatively correlated with lymphocyte numbers in piglets^57^. High levels of Enterobacteriales were also reported to be negatively associated with neutrophilia and to induce a depressed immune response to many common vaccines in infants, including the oral polio virus vaccine, the bacille Calmette-Guérin vaccine against tuberculosis, the tetanus toxoid vaccine, and the hepatitis B vaccine^58^. Thus, it is not surprising that in our study the OTUs annotated to genus *Escherichia-Shigella* and family Enterobacteriaceae were observed with higher relative abundance in individuals that demonstrated weak responses to the IAV vaccine.

Our data enabled us to create a list of 81 OTUs found in the pre-vaccination D28 fecal microbiota that predicted extreme vaccine responders at early (D49), maximum (D56 and D63), and persistent (D146) response stages, based on both HAI titers and IAV-specific IgG levels. In general, predictions were better for IAV-specific IgG response at D63 and D146. This analysis exploited the extent of inter-individual variability in the fecal microbiota before vaccination to explain variations in the intensity of immune response post-vaccination. This variability was observed early in life, and had a significant association with vaccine response throughout their life: from certain combinations of OTUs in the D28 microbiota, we were able to predict the persistence of the IAV-specific IgG response over 100 days later. Although the idea of predicting vaccine response is more often explored in human medicine^59^, our work on the microbiota of pigs suggests that predictions should also be possible in this species. The relevance and feasibility of such an approach in veterinary medicine remain to be discussed and evaluated. Many vaccines are often administered early in life at a time when neither the immune system nor the gut microbiota are very mature^60^. In pigs, the gut microbiota has been shown to stabilize, after an initial phase of diversification, at 40 days of age^36^, but vaccines are often administered at weaning, together with other farm practices that can include the administration of antibiotics to avoid morbidity post-weaning. The importance of the early gut microbiome in the acquisition of an individual’s immune competence led to this period being defined in mice as a ‘critical window’. The present study suggests the likely existence of a similar critical window in pigs, with possible long-term effects, as shown here by the links we observed with the persistence of the vaccine response. At weaning, bacteria from genus *Prevotella* or family Muribaculaceae might favor a strong vaccine response, while some bacteria from the genera *Helicobacter* and *Bacteroides* genus might impair it. The contribution to immune-system maturation or the immunogenicity of bacterial metabolites (e.g., flagellin) or microbe-associated molecular patterns (e.g., LPSs) could be addressed by further study of the bacterial molecular patterns involved.

In conclusion, our results highlight the importance of interactions between the gut microbiota and the immune response to vaccination in pigs. We identified several candidate OTUs present early in life, before vaccination, that may influence the immune response to the IAV vaccine either positively, such as bacteria from genus *Prevotella* or family Muribaculaceae, or negatively, as with bacteria from the genera *Helicobacter* or *Bacteroides*. Our OTU analysis highlighted the need to implement whole-metagenome sequencing to characterize microbial communities to the levels of species and functions. Our results revealed links between bacterial richness in the fecal microbiota and vaccine response, and suggest that farm and breeding practices that support gut bacterial richness should yield benefits for optimizing vaccine efficacy. Our results enabled us to identify associations, but not causalities, between microbiota composition before vaccination and the vaccine response. More investigations are needed to deepen our understanding of this relationship, and specific experimental designs such as fecal microbiota transplantation have to be set up for biological validation. However, our results provide initial insights that might be applied to the identification of candidates for both predicting and improving vaccine efficiency.

## Methods

### Animals and biological sampling

All animal procedures were performed according to guidelines for the care and use of experimental animals (permission for animal experimentation to C. Rogel-Gaillard: A78-172; agreement for experimentation from the Centre de Recherche INRA Poitou-Charentes - Site expérimental du Magneraud: A17661; protocol approved by the French Ministry of Research with authorization ID APAFIS#4295-2016022615583351 v4 after review by ethics committee No. 084).

The experiment was conducted on the INRAE experimental farm at le Magneraud (GenESI, Pig Phenotyping and Innovative Breeding Facility, (10.15454/1.5572415481185847E12). We produced 26 litters of Large White pigs (Supplementary Data 1) by mating 26 different boars with 25 sows, one sow having been inseminated twice. The animals were born in five batches over a period of six months and were grown indoors. Piglets were weaned at D28 on average (D24 to D30). The animals were raised under standard conditions in pens of 20 to 30 animals during the post-weaning period (D28 to D68) and in pens of 10 to 12 animals during the growth period (from around D68 to D146). After weaning, the pigs were fed *ad libitum* with a commercial diet (GUSTI PRIM, NUTRICIAB®) up to D34, then with a post-weaning diet up to D68, and finally with a growth diet until slaughter (see Supplementary Table 4 for details on diets). No antibiotic treatment was administered at any time during the experiment. At weaning, all animals were weighed (Supplementary Data 1) and classified according to three fecal scores: “no diarrhea” (0-regular stool), “intermediate” (1-soft stool), or “diarrhea” (2-liquid stool). On the day of weaning, two males and two females per litter were vaccinated with a commercial inactivated IAV vaccine directed against subtypes H1N1, H3N2, and H1N2 (Respiporc Flu3, IDT Biologika). Three weeks later, at D49, all vaccinated pigs received a booster vaccination. In each litter, one male and one female were not vaccinated and were used as control animals. In total, 131 piglets were included in the study, comprising 98 vaccinated pigs (48 females and 50 males) and 33 non-vaccinated control pigs (17 females and 16 males). Except transitory diarrhea at weaning for eight animals (Supplementary Data 1), no disease outcome was recorded and growth curves were consistent with good health. All pigs were also followed-up with blood cell counts (data not shown) that revealed no obvious health problems either.

Blood was collected at D28, D49, D56, D63, and D146 from the jugular vein of pigs in dry tubes in order to determine humoral vaccine responses in serum. Stool samples were collected at D28, D49, D63, and D146 directly from rectal ampulla, snap-frozen in liquid nitrogen (200 mg per cryotube), and stored at −80 °C until use for microbial DNA extraction. All biological samples were stored at the Biological Resources Center of the @BRIDGe core facility that is a member of the CRB-Anim infrastructure (10.15454/1.5613785622827378E12, CRB-Anim, INRA, 2018. Biological Resource Centers for domestic animals of AgroBRC). A summary of all samples collected at each time is available in Supplementary Table 5.

### Characterization of the vaccine response and identification of groups of high and low responders

Vaccine response was assessed at D28, D49 (21 days post-vaccination; dpv), D56 (28 dpv), D63 (35 dpv), and D146 (118 dpv) by measuring levels of IAV-specific IgG in the serum and performing HAI assays. D49 corresponded to the early vaccine response, D56 and D63 represented the maximum response, and D146 qualified the persistent response.

Briefly, IAV-specific IgG levels were measured with a semi-quantitative ELISA on plates coated with inactivated virus strains that were homologous to the three subtypes targeted by the vaccine: avian-like swine H1avN1 (A/Sw/Cotes d’Armor/0388/09); human-like reassortant swine H3N2 (A/Sw/Spain/SF32071/07); and human-like reassortant swine H1huN2 (A/Sw/Spain/SF12091/07). Sera were diluted 1/100, 1/1000, or 1/10,000 in duplicate. In parallel, a standard curve was constructed using anti-pig IgG–coated plates and a pig reference serum. For detection, HRP-conjugated anti-pig IgG was used in combination with a tetramethylbenzidine substrate. Absorbance at 450 nm was measured, and levels of IAV-specific IgG in sera were extrapolated using the standard curve and expressed in µg/mL or pg/mL.

HAI assays were performed according to standardized procedures^61^. Briefly, 25 µL of two-fold serial dilutions (starting from 1:10) of RDE-inactivated sera (receptor-destroying enzyme, Denka Seiken) were incubated with 25 μL of 4 hemagglutination (HA) assay units of virus inoculum, constituted of a mix of the three strains. Then, 50 µL of Turkey red blood cells (RBC) (0.5% final dilution, Tebu-bio) were added. Wells were examined visually for HA inhibition, as indicated by the appearance of well-defined RBC “buttons” or teardrop formation upon plate tilting. The final HAI titer was the reciprocal of the highest dilution of serum that completely prevented HA.

Animals were identified as high (HR) or low responders (LR) at each time-point post-vaccination according to the ranking of their response levels within the entire population of vaccinated pigs (*n* = 98). At each time point, HR and LR pigs were identified as individuals with IAV-specific IgG levels that were higher than the mean + 1 standard deviation (SD) or less than the mean − 1 SD, respectively (Fig. 1b). For HAI titers, HR and LR groups were defined as follows: at 21 dpv, LR pigs had HAI ≤ 20 and HR pigs had HAI ≥ 160; at 28 and 35 dpv, LR pigs had HAI ≤ 160 and HR pigs had HAI ≥ 640; at 118 dpv, LR pigs had HAI ≤ 10 and HR pigs had HAI ≥ 80 (Fig. 1c). The composition of HR and LR groups is reported in Supplementary Data 1. It differed among post-vaccination time points in order to maintain the selection criteria as defined above.

### Fecal DNA extraction and 16S rRNA sequencing

DNA extraction was performed as previously described^62^. In brief, 200 mg of frozen fecal sample were resuspended with 250 μL of guanidine thiocyanate buffer (4 M guanidine thiocyanate–0.1 M Tris, pH 7.5), 40 μL of 10% *N*-lauroyl sarcosine–0.1 M phosphate buffer (pH 8.0), and 500 μL of 5% *N*-lauroyl sarcosine; the mixture was then incubated at 70 °C for 1 h. After the addition of one volume of 0.1-mm-diameter silica beads (Sigma), tubes were shaken for 10 min at the maximum speed on a Vibrobroyeur MM200 (Retsch, Germany). After shaking, tubes were centrifuged at 20,000 × *g* for 5 min at 4 °C. After recovery of the supernatant, 30 μL of Proteinase K (Chemagic STAR DNA BTS kit, Perkin Elmer, USA) were added; samples were incubated for 10 min at 70 °C at 250 rpm in a Multi-Therm shaker (Benchmark Scientific, USA) and then for 5 min at 95 °C for enzyme inactivation. After centrifugation at 20,000 × *g* for 5 min at 4 °C, DNA extraction was performed on the supernatant using the Chemagic STAR DNA BTS kit (Perkin Elmer, USA) and the Chemagic STAR platform (Hamilton, Perkin Elmer, USA), according to the manufacturer’s instructions.

Amplicon libraries of the V3–V4 region of the 16S rRNA gene were constructed; amplification was performed using the PCR1F_343 (5′-CTTTCCCTACACGACGCTCTTCCGATCTACGGRAGGCAGCAG-3′) and PCR1R_784 (5′-GGAGTTCAGACGTGTGCTCTTCCGATCTTACCAGGGTATCTAATCCT-3’) primers following the Illumina 16S rRNA metagenomic sequencing library preparation protocol. Paired-end sequencing of the pooled library was performed on an Illumina MiSeq platform (Illumina Inc., San Diego, CA, USA) using the Miseq Reagent kit v3 (2 × 300 cycles, Illumina Inc., San Diego, CA, USA), as previously described^32,62^. FastQ files were generated after the run was completed (MiSeq Reporter software, Illumina, USA). The raw sequences are available through accession PRJNA647267 on the NCBI Sequence Read Archive.

### Sequence analysis: identification and quantification of OTUs

We used the FROGS pipeline (v3.0)^63^ to assemble read pairs, to trim the PCR1F_343 and PCR1R_784 primers, and to filter sequences based on length, keeping only amplicons with a length between 300 and 490 base pairs. Unique sequences, with data on their relative abundance per sample, were clustered with FROGS using Swarm (v 1.4.1)^64^, first with an aggregation distance of 1 and then of 3. FROGS removes chimeras using the Vsearch algorithm (v2.6.0)^65^. Rare OTUs were filtered out (abundance <0.005% of total)^66^ and the remaining sequences were annotated via NCBI Blast + (2.6.0)^67^ alignment with 16S rRNA reference sequences from the SILVA database (version 132 from the 2017 update)^68^. Using these data, we obtained an abundance table and a phylogenetic tree (FROGS Tree tool using Mafft (v7.310)^69^ and Fasttree (2.1.10)^70^). Based on the sequencing depth and the rarefaction curves, we rarefied counts for alpha and beta diversity analyses at 10,000 counts per sample. After this rarefaction step, a few samples were excluded; the final dataset contained 89 animals at D28 (*n*_D28_ = 89), 97 animals at D49 (*n*_D49_ = 97), 98 animals at D63 (*n*_D49_ = 98), and 83 animals at D146 (*n*_D49_ = 83) (Supplementary Table 5). Overall, there were 74 animals with data available at all four sampling time points (D28, D49, D63, and D146), with which we studied the evolution of the fecal microbiota from the day of vaccination until slaughter.

### Analysis of fecal microbiota composition and diversity, and association with animal phenotypes

Bio-statistical analyses were performed in R 3.5.2^71^. Using the distributions of OTU counts, we inferred taxonomic composition at the levels of phylum, family, and genus with the package Phyloseq 1.26.1^72^. Alpha diversity was evaluated through calculations of species richness, evenness, Shannon index values, and inverse Simpson index values with the package Vegan 2.5.5^73^. Beta diversity was calculated using Bray-Curtis distances. Vegan was also used to create a non-metric multidimensional scaling (NMDS) representation of Bray-Curtis distances between each extreme response group (defined by IAV-specific IgG or HAI titer).

We first investigated the effects of body weight at weaning, age at weaning, sex, and batch on alpha diversity using maximum-likelihood ratio tests. We then investigated the association of these factors with beta diversity using Adonis model based on Bray-Curtis distances (package Vegan).

To investigate the association between alpha diversity at D28, D49, and D63 on the contemporaneous and subsequent vaccine responses, we implemented a linear mixed-effect model of each alpha-diversity indicator (Richness, Shannon) and the numerical value of Log10 IAV-specific IgG and Log2 HAI titers, considering all available data at each time point (nlme package, 3.1-142)^74^. In these models, the weaning age, sex, and batch were set as co-variables because they all had a significant association with the vaccine response. The litter was set as a random effect.

We also investigated the association between fecal microbiota composition at D28, D49, and D63 and the subsequent vaccine response, by considering only the extreme responders for each phenotype (IAV-specific IgG or HAI titers). At D28, D49, and D63, we applied Adonis models to test dissimilarites between the fecal microbiota of pigs classified as HR or LR for IAV-specific IgG or HAI titers at later times points (package Vegan). For these models, sex, batch, and weaning age were set as co-variables.

Previous research identified two enterotypes in 60-day-old Large White pigs, each driven by two genera (*Prevotella* and *Mitsuokella* or *Treponema* and *Ruminococcus*)^75^. Following a similar approach, we determined individual enterotypes with the Jensen-Shannon divergence distance calculated from the genus abundance table^76^. To determine whether the enterotypes we observed were associated with vaccine response levels (measured either by IAV-specific IgG levels or HAI titers), we performed a chi-squared test that considered the number of pigs in each enterotype group and in each extreme responder group.

### Comparisons of HR and LR groups: differential abundance of OTUs and pairwise correlations

For the animals that demonstrated extreme vaccine responses (HR or LR for IAV-specific IgG levels or HAI titers) at D49, D56, D63, and D146, we analyzed the pre-vaccination microbiota (D28) with the R package metagenomeSeq 1.24.1^77^ to identify OTUs that displayed differential abundance among groups. This package enables the normalization of raw sequence counts through a cumulative-sum scaling method to reduce biases due to uneven sequencing depth^78^. We used a zero-inflated Gaussian mixture model with vaccine response as the main effect, and sex, weaning age, and batch as co-variables, and applied an FDR adjustment of 0.05. MetagenomeSeq was also used to study whether the set of OTUs that were differentially abundant at D28 between groups that later demonstrated high or low vaccine response (at D56 and D63) maintained these same patterns of differential abundance in the fecal microbiota of the same animals at older ages (D49 and D63). For the same set of OTUs, we also determined the occurrence of each at D28, D49, D63, and D146 in the fecal microbiota of each member of the larger population (*n* = 89). For each OTU, we also counted all other OTUs that were annotated to the same genus in the D28 fecal microbiota of the 89 vaccinated piglets.

To identify mutually excluding or co-abundant OTUs, we calculated pairwise correlations between all OTUs in the overall dataset at D28 (*n* = 89), using the bootstrap method from fastspar software^79^. We specifically looked at correlations between the 23 OTUs that were identified as differentially abundant between the high and low extreme responders for IAV-specific IgG at D63. Moderate and high correlations were included in a network representation, using a cutoff of 0.4 and 0.5 for the correlation coefficient and of 0.05 for the *p*-value, with the R packages igraph and network^80^.

### Sparse partial least squares discriminant analysis (sPLS-DA), PLS-DA, and PLS analysis

We searched for OTUs that were predictive of extreme responses for IAV-specific IgG and HAI titers (LR versus HR) at the different time points using sparse partial least squares discriminant analysis (sPLS-DA). OTUs were normalized using cumulative sum square normalization with the metagenomeSeq package, and sPLS-DA was performed using the MixOmics package^81^. Prediction accuracy was estimated through calculations of the balanced error rate (BER), which measures the rate of overall sample misclassification; the lower the BER, the more reliable the prediction. The minimal number of components to keep was chosen based on barplots of eigenvalues. We then tuned each sPLS-DA, in two steps, to determine the number of OTUs to keep for each component. We first estimated this number by considering a set of OTUs that varied from 1 to 200 in steps of 5, using a fivefold validation process repeated 100 times. From the plot of BER evolution, we recorded the optimum number of OTUs to retain for each component. We then tuned the sPLS-DA again, but this time considered a set of OTUs that varied from 1 up to this first optimum number, in steps of 1. The final number of OTUs retained corresponded to the number observed just before the BER prediction reached a plateau. We then searched for the OTU with the largest contribution to each type of extreme vaccine response. To evaluate the ability of each sPLS-DA classification to predict the vaccine response level, we calculated their corresponding BER, p-value, and area under the curve (AUC). Then, in an attempt to improve the prediction performance, we merged all OTUs identified by sPLS-DA and applied a PLS-DA to the two groups of extreme responders. Finally, we carried out a PLS analysis on the IAV-specific IgG levels and HAI titers from the whole population of 89 piglets for which we had fecal microbiota data at D28. We assessed the confidence of the prediction and calculated the Q2 (‘goodness of prediction’) parameter to determine whether it was below the recommended limit of 0.0975. We measured the Pearson correlation coefficient between the predicted and measured levels of IAV-specific IgG, and we performed a Shapiro-Wilcoxon test to assess the normality of the residuals of the differences between predicted and measured values.

### Reporting summary

Further information on research design is available in the Nature Research Reporting Summary linked to this article.

## Supplementary information

Supplementary Information

Supplementary Data 1-9

Reporting Summary

## Data Availability

The raw sequences are available through accession PRJNA647267 on the NCBI Sequence Read Archive. Metadata on animal rearing, phenotype, and vaccine response are available in Supplementary Data 1. The datasets generated and/or analyzed during the current study are available from the corresponding author on reasonable request.
