# Supplementary material for: Links between fecal microbiota and the response to vaccination against influenza A virus in pigs
Source: NPJ Vaccines. 2021 Jul 22;6:92. doi: 10.1038/s41541-021-00351-2 (PMC8298503; doi:10.1038/s41541-021-00351-2)
Supplement: Supplementary file 1 — Supplementary Information [file 41541_2021_351_MOESM1_ESM.pdf]

Supplementary Information

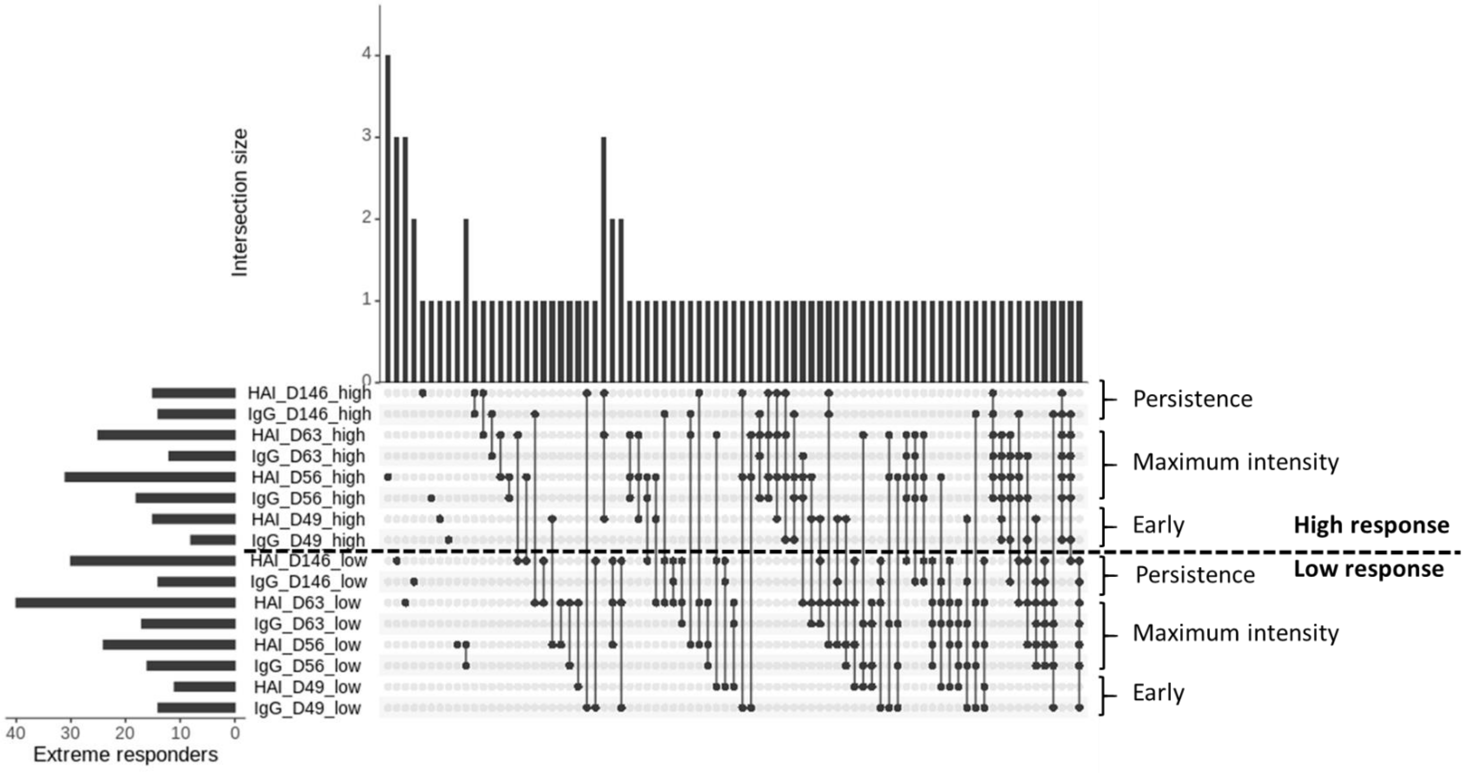

Supplementary Figure 1: Matrix layout for all intersections of extreme responders for IAV-specific IgG levels and HAI titers at D49, D56, D63 and D146. Dark circles in the matrix indicate sets that are part of the intersection. The black bars on the left side of the figure indicate the total number of extreme high or low responders per group. The dotted line separates the two sets of high and low responder groups into subsets corresponding to early response (49 days of age on the boost day, three weeks after the first vaccine injection), the maximum intensity response at 56 and 63 days of age (one and two weeks after the boost) and the persistence of Ab response at 146 days of age (118 days post-vaccination).

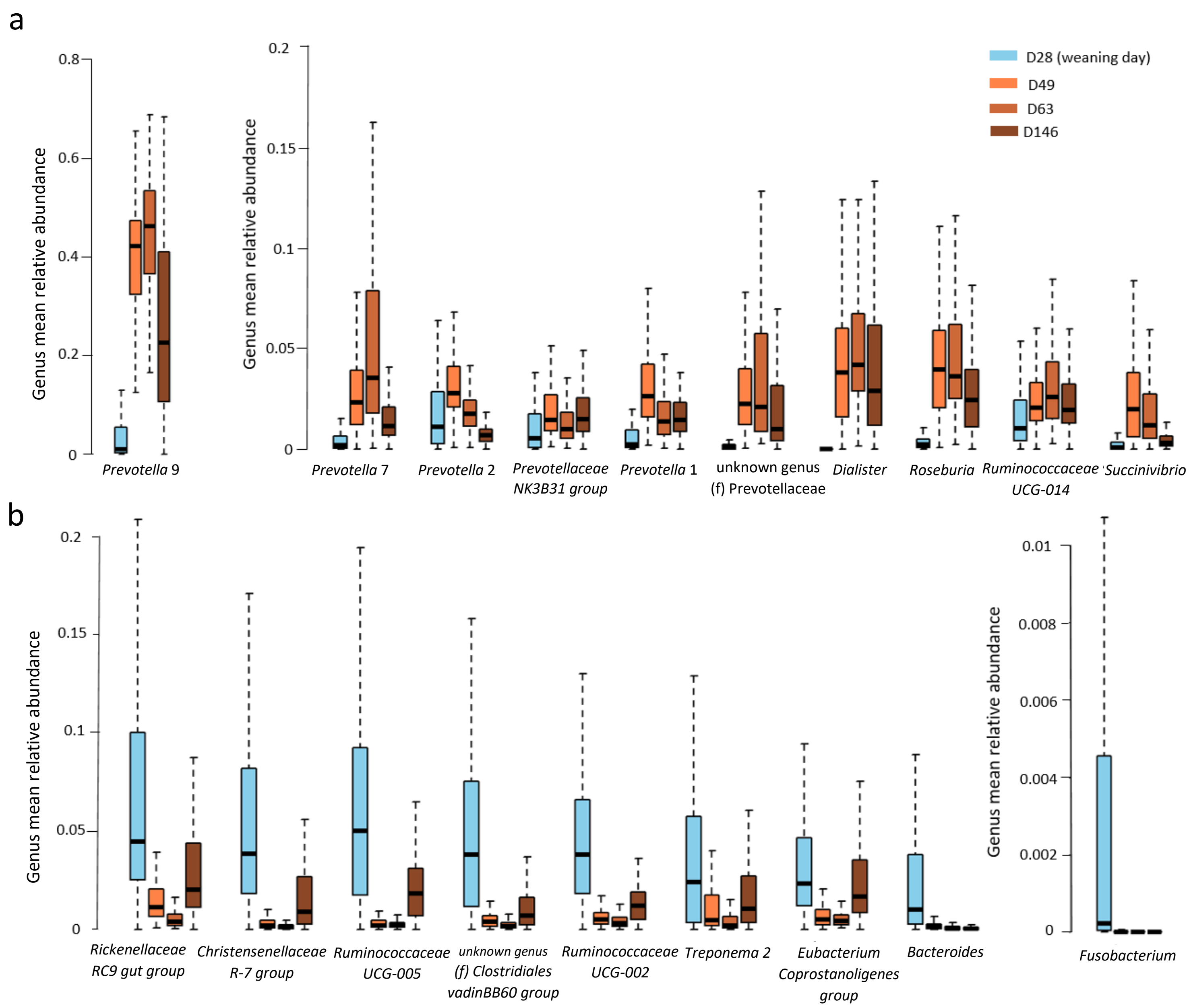

Supplementary Figure 2: Evolution of the relative abundance of the main genera of fecal microbiota from D28 to D146. The relative abundances of all genera are represented by boxplots colored according to animal age; blue at D28 and an orange gradient from pale to dark for D49, D63, and D146. **a** Relative abundances of the ten main bacterial genera observed at D63, with their corresponding abundances at D28, D49, and D146. **b** Relative abundances of the nine main bacterial genera observed at D28, with their corresponding abundances at D49, D63, and D146. *Prevotella 9*, as the most abundant genus in the D63 fecal microbiota and the eighth most abundant genus in the D28 fecal microbiota, is represented only once. Corresponding data are available in Supplementary Data 3.

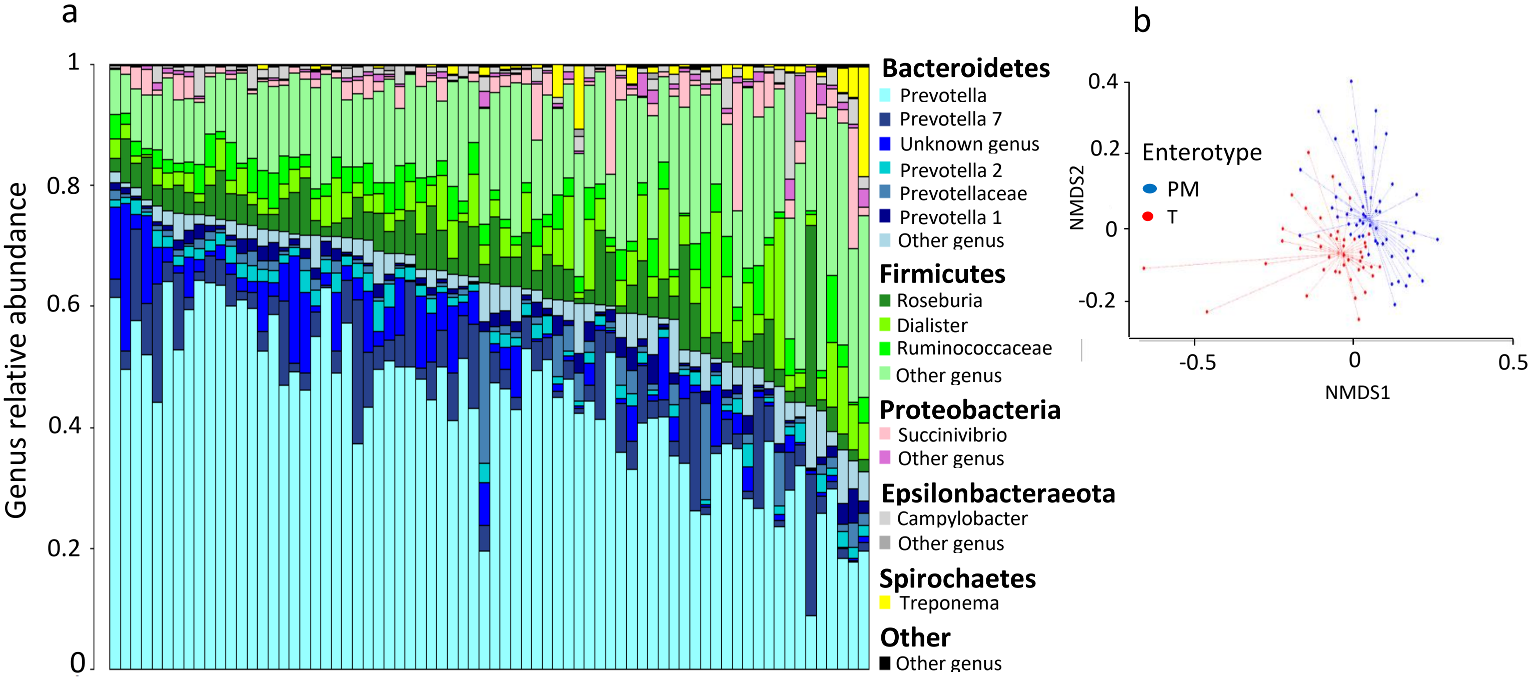

Supplementary Figure 3. Composition of fecal microbiota of vaccinated pigs at D63 and visualization of clustering according to two enterotypes. a Relative abundances of bacterial genera in the fecal microbiota of 74 vaccinated pigs in which the composition of the fecal microbiota was followed from D28 to D146 (slaughter). Only genera with mean abundances over 1.8% are represented, with the exception of *Campylobacter* and *Treponema 2*. **b** Distribution of pigs between the enterotypes PM (*Prevotella-Mitsuokella*) and T (*Treponema*). Animals corresponding to enterotypes PM and T are represented by blue and red dots, respectively. All data are available in Supplementary Table 2 and in Supplementary Data 3.

a

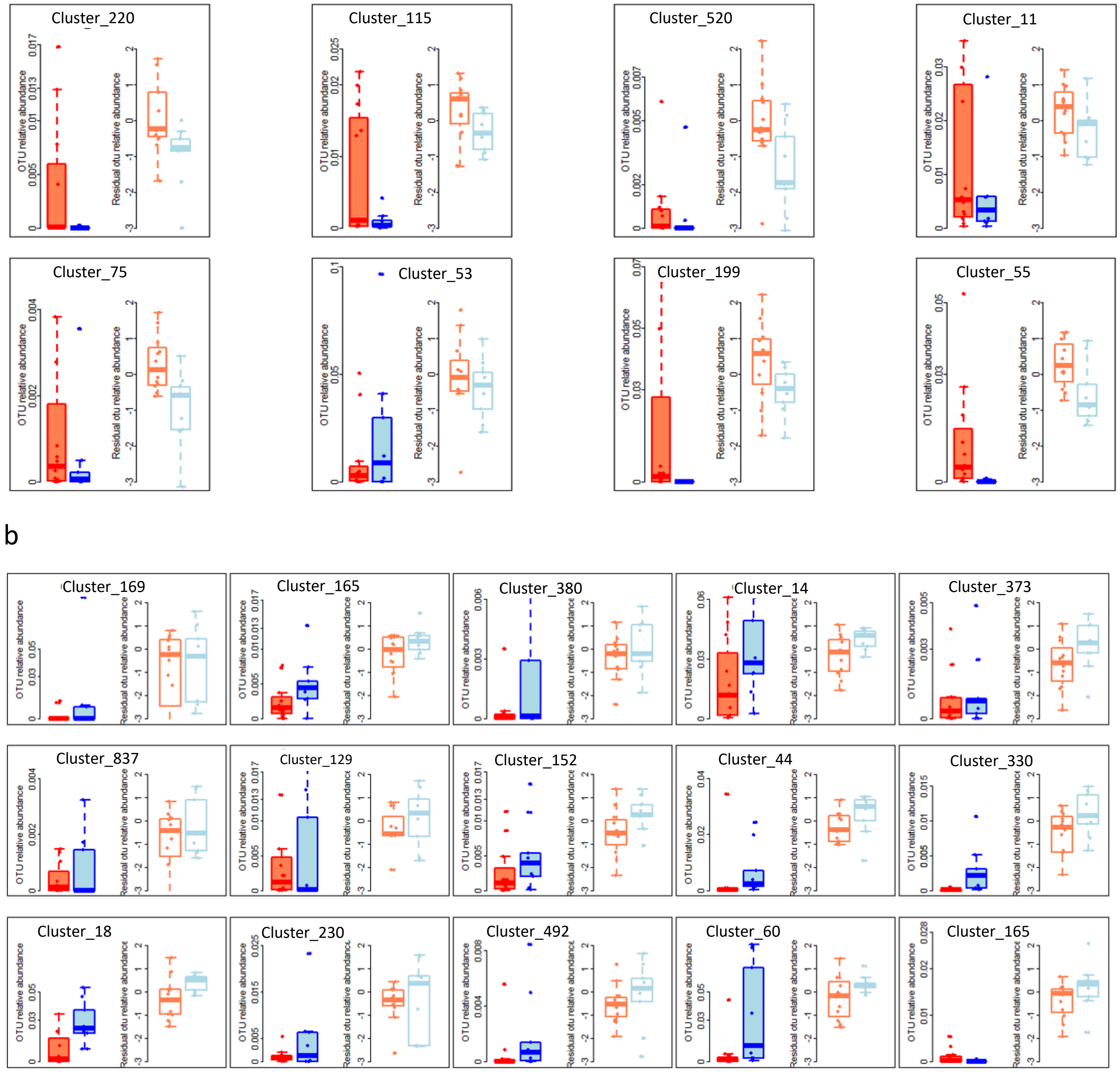

Supplementary Figure 4. Details on the differential abundance of OTUs in the D28 fecal microbiota of high and low vaccine responders, measured by serum levels of IAV-specific IgG at D63. a Differential abundance of OTUs that were more abundant in high responders. **b** Differential abundance of OTUs that were more abundant in low responders. For each OTU, the first boxplot displays its relative abundance in the high (red) and low (blue) responders. The second boxplot displays the same data with normalization by metagenomeSeq package of the OTU counts for gender, weaning age, and batch, as these three variables had a significant effect on IAV-specific IgG levels and were consequently set as fixed effects in the model. All data are available in Supplementary Data 6.

Supplementary Table 1: Association between pig characteristics and diversity indices of OTUs in fecal DNA at D28 for the group of vaccinated pigs.

Age (day)	Zootechnical parameters	Alpha diversity		Beta diversity
		Richness	Shannon	
D28	Body weight at weaning	**0.0031****	**8.0 x 10^{-4}****	**0.0096****
	Batch	0.9638	0.8874	**0.0016****
	Age at weaning	0.8816	0.2691	**0.0001****
	Sex	0.4511	0.4134	0.5542

P-values are reported in the table and highlighted in bold letters where significant (* for p-value <0.05, and **for p-value<0.01). The effect of each zootechnical parameter on the alpha diversity was measured on the total vaccinated population using maximum likelihood ratio tests on linear models. Individual alpha diversity indices are available in Supplementary Data 5 for all animals. The effect of the beta diversity was measured with Bray-Curtis distances, and p-values were calculated with an Adonis test.

Supplementary Table 2: Summary of differentially abundant genera between the two fecal DNA enterotypes found at D63.

Enterotype	Phylum	Family	Genus	Log2 FC enterotype	p-value	FDR	Mean relative abundance	
							PM	T
PM	**Bacteroidetes**	**Prevotellaceae**	**Prevotella 7**	**-2.2**	**6.8E-11**	**9.5E-09**	**3.4E-01**	**8.0E-02**
	**Firmicutes**	**Veillonellaceae**	**Mitsuokella**	**-2.2**	**4.9E-10**	**3.4E-08**	**9.0E-02**	**1.7E-02**
	Firmicutes	Veillonellaceae	Multi-affiliation	-2.0	9.1E-07	3.1E-05	6.4E-03	1.1E-03
	Proteobacteria	Succinivibrionaceae	Succinivibrionaceae UCG-001	-1.8	5.4E-03	3.0E-02	1.1E-02	6.0E-04
	Proteobacteria	Succinivibrionaceae	Succinivibrio	-1.4	1.5E-03	1.1E-02	1.4E-01	3.8E-02
	Firmicutes	Streptococcaceae	Streptococcus	-1.1	4.2E-03	2.6E-02	2.8E-02	1.3E-02
	Firmicutes	Ruminococcaceae	Flavonifractor	-1.0	3.5E-03	2.2E-02	1.2E-04	3.6E-05
	Proteobacteria	Succinivibrionaceae	Anaerobiospirillum	-0.9	3.5E-03	2.2E-02	9.7E-04	3.7E-04
	Firmicutes	Veillonellaceae	Dialister	-0.9	6.0E-04	6.5E-03	2.6E-01	1.2E-01
	Actinobacteria	Atopobiaceae	Olsenella	-0.7	7.1E-03	3.7E-02	3.0E-04	1.5E-04
	Firmicutes	Lachnospiraceae	Lachnoclostridium	-0.7	1.6E-03	1.1E-02	1.8E-03	1.2E-03
	Firmicutes	Erysipelotrichaceae	Holdemanella	-0.6	1.6E-03	1.1E-02	7.9E-04	3.9E-04
	Firmicutes	Ruminococcaceae	Ruminiclostridium	-0.6	1.2E-02	4.9E-02	2.0E-04	2.2E-04
	Bacteroidetes	Prevotellaceae	Prevotella 9	-0.6	9.0E-04	7.9E-03	1.7E+00	1.3E+00
T	**Spirochaetes**	**Spirochaetaceae**	**Treponema 2**	**1.1**	**9.3E-03**	**4.3E-02**	**9.1E-03**	**3.7E-02**
	Firmicutes	Lachnospiraceae	Anaerosporobacter	3.0	5.5E-07	2.5E-05	3.6E-06	9.5E-04
	Firmicutes	Lachnospiraceae	Shuttleworthia	2.9	3.2E-05	6.4E-04	1.2E-02	6.2E-02
	Bacteroidetes	p-2534-18B5 gut group	unknown genus	2.1	4.2E-05	7.0E-04	2.8E-04	5.8E-03
	Firmicutes	Lachnospiraceae	Lachnospiraceae AC2044 group	1.5	4.5E-05	7.0E-04	1.8E-05	5.5E-04
	Firmicutes	Erysipelotrichaceae	Turicibacter	1.4	1.7E-06	4.8E-05	4.2E-05	1.5E-04
	Bacteroidetes	Prevotellaceae	Prevotellaceae NK3B31	1.3	5.1E-05	7.1E-04	2.6E-02	8.1E-02
	Firmicutes	Lachnospiraceae	unknown genus	1.3	1.4E-05	3.2E-04	1.0E-02	2.2E-02
	Firmicutes	Lachnospiraceae	Lachnospiraceae UCG-003	1.2	8.8E-05	1.1E-03	1.2E-04	7.0E-04
	Bacteroidetes	Paludibacteraceae	unknown genus	1.1	9.7E-03	4.3E-02	1.9E-06	3.9E-04
	Bacteroidetes	Prevotellaceae	Prevotella	1.1	7.8E-04	7.8E-03	8.8E-05	6.5E-04
	Firmicutes	Veillonellaceae	Anaerovibrio	1.1	5.7E-03	3.0E-02	2.2E-03	7.3E-03
	Firmicutes	Peptostreptococcaceae	Multi-affiliation	1.0	1.4E-04	1.6E-03	1.0E-05	1.1E-04
	Firmicutes	Lachnospiraceae	Agathobacter	1.0	1.0E-03	8.1E-03	8.8E-03	1.5E-02
	Firmicutes	Lachnospiraceae	[Eubacterium] xylanophilum group	0.9	5.0E-03	2.9E-02	1.1E-03	2.5E-03
	Bacteroidetes	Prevotellaceae	Prevotella 1	0.8	9.0E-04	7.9E-03	3.3E-02	6.8E-02
	Firmicutes	Lachnospiraceae	Marvinbryantia	0.7	7.6E-03	3.8E-02	9.2E-04	1.8E-03
	Bacteroidetes	F082	unknown genus	0.7	9.0E-03	4.3E-02	1.3E-05	5.7E-05
	Firmicutes	Ruminococcaceae	Pygmaiobacter	0.5	1.0E-02	4.5E-02	2.3E-05	8.7E-05

The enterotype referred to as PM is more abundant in OTUs annotated to the *Prevotella* and *Mitsuokella* genera. The enterotype referred to as T is more abundant in OTUs annotated to the *Treponema* genus.

Supplementary Table 3: Summary of the fecal microbiota OTU at D49, D63, and D146 that were found significantly differentially abundant between high and low responders to the vaccine measured by levels of seric IAV-specific IgG and haemagglutination inhibition assay at D49, D56, D63, and D146.

Microbial community	Vaccine response phenotype	OTU	Taxonomic annotation			Log2 FC	p-value	FDR	Mean relative abundance	
			Phylum	Family	Genus				HR[2]	LR[3]
D49	IAV specific-IgGs at D49	Cluster_125	Firmicutes	Peptostreptococcaceae	Terrisporobacter	-14.77	4.27E-04	0.0287	0.00081	0.00149
		Cluster_490	Firmicutes	Lachnospiraceae	Lachnospiraceae FCS020 group	-3.83	2.89E-04	0.0277	0.00050	0.00033
		Cluster_409	Firmicutes	Ruminococcaceae	[Eubacterium] coprostanoligenes group	3.32	5.69E-04	0.0318	0.00018	0.00047
		Cluster_693	Firmicutes	Ruminococcaceae	Ruminococcaceae UCG-014	3.25	9.60E-04	0.0496	0.00009	0.00080
		Cluster_158	Bacteroidetes	Prevotellaceae	Alloprevotella	-4.35	2.27E-06	0.0010	0.00363	0.00119
		Cluster_178	Bacteroidetes	Prevotellaceae	Prevotellaceae NK3B31 group	-4.10	3.00E-06	0.0010	0.00346	0.00145
		Cluster_120	Bacteroidetes	Prevotellaceae	Prevotellaceae NK3B31 group	-3.84	1.06E-05	0.0024	0.00553	0.00207
		Cluster_559	Bacteroidetes	Prevotellaceae	Prevotellaceae NK3B31 group	-3.51	1.84E-04	0.0212	0.00075	0.00033
		Cluster_372	Bacteroidetes	Prevotellaceae	Prevotella 9	-3.65	1.09E-04	0.0183	0.00205	0.00105
		Cluster_307	Bacteroidetes	Prevotellaceae	Prevotella 9	-2.91	3.89E-04	0.0287	0.00157	0.00065
		Cluster_2515	Bacteroidetes	Prevotellaceae	Prevotella 9	-3.15	5.51E-04	0.0318	0.00051	0.00022
		Cluster_844	Bacteroidetes	Prevotellaceae	Prevotellaceae NK3B31 group	-3.08	3.31E-04	0.0278	0.00131	0.00048
		Cluster_13	Firmicutes	Lactobacillaceae	Lactobacillus	3.06	1.90E-04	0.0212	0.01277	0.01534
	HAI[1] at D56	Cluster_620	Firmicutes	Ruminococcaceae	[Eubacterium] coprostanoligenes group	2.28	8.93E-04	0.0496	0.00028	0.00040
		Cluster_159	Spirochaetes	Spirochaetaceae	Treponema 2	2.20	6.62E-04	0.0496	0.00080	0.00248
		Cluster_602	Firmicutes	Ruminococcaceae	Ruminococcaceae UCG-014	2.05	4.31E-04	0.0496	0.00012	0.00032
		Cluster_857	Firmicutes	Ruminococcaceae	unknown genus	1.82	9.18E-04	0.0496	0.00013	0.00021
		Cluster_592	Bacteroidetes	Muribaculaceae	unknown genus	1.81	1.23E-03	0.0496	0.00025	0.00063
		Cluster_323	Bacteroidetes	Prevotellaceae	Prevotella 7	1.81	9.00E-04	0.0496	0.00010	0.00044
		Cluster_115	Firmicutes	Ruminococcaceae	Ruminococcaceae UCG-005	1.74	5.47E-05	0.0296	0.00006	0.00016
		Cluster_219	Bacteroidetes	Prevotellaceae	Prevotella 9	-1.70	1.17E-03	0.0496	0.00048	0.00018
		Cluster_345	Firmicutes	Lachnospiraceae	Multi-affiliation	-1.67	5.07E-04	0.0496	0.00046	0.00021
		Cluster_735	Firmicutes	Ruminococcaceae	Ruminococcaceae UCG-014	1.66	3.63E-04	0.0496	0.00005	0.00017
		Cluster_1391	Bacteroidetes	Prevotellaceae	Prevotella 9	-1.60	3.24E-05	0.0296	0.00007	0.00001
		Cluster_70	Bacteroidetes	Prevotellaceae	Prevotella 7	1.52	9.90E-04	0.0496	0.00067	0.00255
		Cluster_960	Firmicutes	Ruminococcaceae	Ruminococcus 1	1.42	1.01E-03	0.0496	0.00007	0.00013
		Cluster_2268	Bacteroidetes	Prevotellaceae	Alloprevotella	-1.38	3.54E-04	0.0496	0.00017	0.00006
		Cluster_113	Firmicutes	Ruminococcaceae	Ruminococcaceae NK4A214 group	-1.36	5.61E-04	0.0496	0.00004	0.00002
		Cluster_673	Firmicutes	Ruminococcaceae	Ruminiclostridium 6	1.35	1.33E-03	0.0496	0.00012	0.00024
		Cluster_648	Bacteroidetes	Prevotellaceae	Prevotella 9	-1.35	4.13E-04	0.0496	0.00009	0.00002
		Cluster_495	Bacteroidetes	Prevotellaceae	Prevotella 9	1.35	8.16E-04	0.0496	0.00016	0.00020
		Cluster_630	Bacteroidetes	Prevotellaceae	unknown genus	-1.35	1.14E-03	0.0496	0.00014	0.00006
		Cluster_888	Firmicutes	Family XIII	Family XIII AD3011 group	-1.34	4.99E-04	0.0496	0.00007	0.00001
		Cluster_262	Firmicutes	Lachnospiraceae	[Eubacterium] eligens group	-1.32	1.11E-03	0.0496	0.00139	0.00079
		Cluster_554	Firmicutes	Christensenellaceae	Christensenellaceae R-7 group	1.26	7.66E-04	0.0496	0.00006	0.00009
		Cluster_662	Bacteroidetes	Muribaculaceae	unknown genus	1.23	1.26E-03	0.0496	0.00034	0.00038
		Cluster_1522	Bacteroidetes	Prevotellaceae	unknown genus	-1.20	1.12E-03	0.0496	0.00008	0.00002
		Cluster_829	Firmicutes	Veillonellaceae	Dialister	-1.17	5.97E-04	0.0496	0.00017	0.00009
		Cluster_2528	Firmicutes	Ruminococcaceae	Ruminococcaceae UCG-014	-1.05	1.28E-03	0.0496	0.00008	0.00007
		Cluster_594	Bacteroidetes	Prevotellaceae	Prevotella 9	-1.02	6.06E-04	0.0496	0.00039	0.00025
		Cluster_37	Firmicutes	Lachnospiraceae	Lachnoclostridium	0.96	1.17E-03	0.0496	0.00006	0.00012
		Cluster_1138	Firmicutes	Ruminococcaceae	Ruminococcaceae UCG-010	0.83	1.23E-03	0.0496	0.00001	0.00004
	HAI at D63	Cluster_18	Bacteroidetes	Rikenellaceae	Rikenellaceae RC9 gut group	-2.45	2.30E-06	0.0021	0.00242	0.00028
		Cluster_561	Firmicutes	Lachnospiraceae	Shuttleworthia	-1.66	6.55E-05	0.0180	0.00016	0.00003
		Cluster_406	Epsilonbacteraeota	Campylobacteraceae	Campylobacter	1.65	2.48E-04	0.0418	0.00003	0.00036
		Cluster_851	Firmicutes	Lachnospiraceae	Shuttleworthia	-1.64	2.09E-04	0.0418	0.00019	0.00003
		Cluster_113	Firmicutes	Ruminococcaceae	Ruminococcaceae NK4A214 group	-1.52	3.77E-06	0.0021	0.00005	0.00002
		Cluster_1037	Firmicutes	Clostridiales vadinBB60 group	unknown genus	1.46	2.66E-04	0.0418	0.00001	0.00009
		Cluster_357	Bacteroidetes	Muribaculaceae	unknown genus	-1.23	2.31E-05	0.0085	0.00005	0.00002

[1] HAI: haemagglutination inhibition assay

[2] HR: High responder [3] LR: Low responder

Microbial community	Vaccine response phenotype	OTU	Taxonomic annotation			Log2 FC	p-value	FDR	Mean relative abundance	
			Phylum	Family	Genus				HR	LR
D63	HAI at D63	Cluster_493	Bacteroidetes	Prevotellaceae	unknown genus	3.72	5.24E-05	0.0183	0.00003	0.00029
		Cluster_963	Firmicutes	Lachnospiraceae	Anaerosporobacter	-3.26	2.31E-06	0.0012	0.00046	0.00011
		Cluster_1007	Bacteroidetes	Prevotellaceae	Prevotella 1	2.65	1.35E-06	0.0012	0.00003	0.00015
		Cluster_1070	Firmicutes	Lachnospiraceae	Fusicatenibacter	-2.00	7.75E-05	0.0203	0.00022	0.00008
		Cluster_722	Firmicutes	Ruminococcaceae	Multi-affiliation	-1.62	2.00E-04	0.0301	0.00018	0.00014
		Cluster_370	Bacteroidetes	Prevotellaceae	Prevotellaceae UCG-001	1.28	2.01E-04	0.0301	0.00002	0.00010
		Cluster_113	Firmicutes	Ruminococcaceae	Ruminococcaceae NK4A214 group	-1.18	1.22E-04	0.0255	0.00022	0.00009
	HAI at D146	Cluster_1026	Bacteroidetes	Prevotellaceae	Prevotella 7	3.13	5.80E-05	0.0287	0.00009	0.00036
		Cluster_904	Bacteroidetes	Prevotellaceae	Prevotella 7	2.52	1.29E-04	0.0424	0.00008	0.00045
		Cluster_510	Bacteroidetes	Prevotellaceae	Prevotellaceae UCG-003	-1.80	1.21E-05	0.0119	0.00027	0.00013
D146	IAV specific-IgGs at D146	Cluster_208	Bacteroidetes	Prevotellaceae	Prevotella 9	-4.14	3.50E-06	0.0026	0.00070	0.00012
		Cluster_416	Firmicutes	Lachnospiraceae	Multi-affiliation	-3.33	1.69E-04	0.0240	0.00121	0.00222
		Cluster_284	Bacteroidetes	Muribaculaceae	unknown genus	-3.25	5.42E-06	0.0026	0.00439	0.00025
		Cluster_66	Spirochaetes	Spirochaetaceae	Treponema 2	-3.22	7.90E-06	0.0026	0.00586	0.00916
		Cluster_725	Firmicutes	Lachnospiraceae	unknown genus	-2.82	4.31E-04	0.0428	0.00118	0.00029
		Cluster_1213	Firmicutes	Streptococcaceae	Streptococcus	2.65	1.04E-05	0.0026	0.00028	0.00136
		Cluster_43	Bacteroidetes	Muribaculaceae	unknown genus	-2.64	1.48E-04	0.0240	0.01922	0.00134
		Cluster_900	Firmicutes	Lactobacillaceae	Lactobacillus	2.56	2.01E-04	0.0250	0.00011	0.00065
		Cluster_183	Firmicutes	Clostridiaceae 1	Sarcina	-2.42	3.04E-04	0.0336	0.00096	0.00029
		Cluster_13	Firmicutes	Lactobacillaceae	Lactobacillus	2.32	1.00E-04	0.0199	0.00331	0.00966
	HAI at D146	Cluster_1114	Firmicutes	Ruminococcaceae	Ruminococcaceae UCG-008	-1.57	9.12E-06	0.0056	6.68E-05	1.89E-04
		Cluster_405	Firmicutes	Ruminococcaceae	Ruminococcaceae UCG-014	1.39	2.49E-05	0.0093	4.59E-04	2.54E-04
		Cluster_1096	Firmicutes	Ruminococcaceae	unknown genus	1.25	8.18E-05	0.0178	3.43E-04	5.34E-05
		Cluster_367	Firmicutes	Lachnospiraceae	Lachnospiraceae NK4A136 group	-1.18	9.51E-05	0.0178	6.93E-05	3.26E-04
		Cluster_304	Bacteroidetes	Prevotellaceae	Prevotellaceae NK3B31 group	-1.08	4.73E-04	0.0475	9.68E-05	3.64E-04
		Cluster_1253	Bacteroidetes	Prevotellaceae	Prevotellaceae NK3B31 group	-1.03	2.24E-04	0.0285	4.51E-05	2.21E-04
		Cluster_220	Firmicutes	Ruminococcaceae	Ruminococcus 1	-1.01	1.60E-04	0.0257	2.07E-04	1.37E-03
		Cluster_753	Firmicutes	Ruminococcaceae	unknown genus	-0.95	1.00E-05	0.0056	9.49E-05	2.01E-04
		Cluster_3017	Firmicutes	Ruminococcaceae	Ruminococcaceae UCG-014	0.84	2.66E-04	0.0299	2.39E-04	3.18E-05
		Cluster_1043	Bacteroidetes	Prevotellaceae	Prevotella 2	-0.80	2.28E-04	0.0285	4.99E-05	1.08E-04
		Cluster_2515	Bacteroidetes	Prevotellaceae	Prevotella 9	-0.76	5.06E-04	0.0475	2.14E-05	6.01E-05
		Cluster_1778	Bacteroidetes	Prevotellaceae	Prevotella 2	-0.71	4.11E-05	0.0116	2.17E-05	7.28E-05

Supplementary Table 4: Composition of pig diets during the initial, post-weaning and growing periods.

	STARTING<br>from 28 to 34 days of age	POST-WEANING<br>from 35 to 68 days of age	GROWING<br>from 69 days of age up to slaughtering
Granulometry (mm)	2.2	2.8	4
Ingredients (% dry matter feed)			
Barley	30.10	25.00	10.00
Cereal mixture	12.3	-	-
Wheat	-	38.35	39.89
Triticale	-	5.00	15.00
Corn	-	-	8.20
CELTI 50% [1]	49.95		
Wheat bran	-	1.8	-
Corn dregs	-	-	2.0
Rapeseed meal	-	-	9.5
Soybean meal 48	2.3	16.30	10.10
Expelled rapeseed meal	-	5.00	-
Toasted soybean	-	2.00	-
Calcium carbonate	-	0.41	0.78
Dicalcium phoshate	-	0.59	-
Salt	-	0.32	0.30
Dextrose	1.50		
Sodium bicarbonate	-	0.08	0.10
Cane molasses	1.00	-	1.5
Palm oil	0.8	0.2	-
Rapeseed oil	0.5	1.50	-
Methionine (liquid)	-	0.2	-
Methionine	-	-	0.03
L-LYSINE 50%	-	0.85	0.46
Choline (liquide)	0.05	-	0.05
Threonine	-	0.40	0.13
Bentonite clay (SMECTAGRI)[2]	-	1.00	1.00
Phytase (BELFEED)[2]	-	0.20	0.20
Phytase (NATUPHOS 5)[2]	-	0.30	0.26
PROPHORCE AC600	0.5	-	-
AL 425 Digesta'Co [2,3]	0.5	-	-
VEVOVITALL [2,4]	0.5	-	-
CMV Post Weaning [2,5]	-	0.50	-
CMV Growing 0.5% [2,5]	-	-	0.50
Nutrients (% Dry matter feed)			
Crude fiber	3.3	4.15	3.86
Raw ash	6.5	5.76	5.55
Crude protein	17.5	17.49	16.03
Crude fat	6.0	4.03	1.82
Phosphorus	0.7	0.50	0.41
Sodium	0.2	0.16	0.16
Calcium	1.1	undetermined	undetermined
Chlorine/chloride	undetermined	0.26	0.28
Met tot	0.6	0.42	0.29
Met + Cys tot	undetermined	0.76	0.63
Lysine	1.5	1.37	1.01
Linoleic acid	undetermined	1.17	0.87
Digestible Energy (ED) (kcal)	undetermined	3274.00	3193.59
Energy (kcal)	2718.6	2350.83	2319.67

[1] blend of cooked wheat, wheat, soybean, cooked soybean, whey, soybean meal, coconut oil, calcium carbonate, potato protein, wheat extract, milk powder, dextrose, monocalcium phosphate, sodium chloride, vitamin A, D3, E, C, trace elements (Fe, I, Cu, Mn, Zn, Se, selenomethionine), food preservative (E324, E310), and dietary supplement (E1606, E954ii, 4a6, and 2b959).

[2] dieatary supplement

[3] Blend of calcium carbonate with wheat

[4] benzoic acid (food preservative)

[5] CMV multivitamin supplement

Supplementary Table 5: Number of vaccinated and non-vaccinated pigs at all experimental time-points and associated data.

	Age in days (D)	Vaccine response step	Pigs	Fecal samples		seric IAV-specific IgG data				HAI[2] data			
				collected	exploited sequencing data[1]	D49	D56	D63	D146	D49	D56	D63	D146
Vaccinated pigs	D28	Before vaccination	98	92	89	89	88	86	80	84	88	86	78
	D49	Early (before booster vaccination)	98	98	97	97	96	94	88	92	96	94	86
	D56	Maximum intensity (after booster)	-	-	-	98	98	95	89	93	97	95	88
	D63	Maximum intensity (after booster)	98	98	98	98	97	95	89	93	97	95	87
	D146	Persistence (before slaughtering)	83	83	83	83	82	81	83	78	82	80	81
Non-vaccinated pigs	D28	Before vaccination	33	33	29	-	-	-	-	-	-	-	-
	D49	-	33	33	33	-	-	-	-	-	-	-	-
	D56	-	-	-	-	-	-	-	-	-	-	-	-
	D63	-	33	33	33	-	-	-	-	-	-	-	-
	D146	-	33	30	30	-	-	-	-	-	-	-	-

[1] Number of samples kept after the rarefaction step of the fecal DNA sequencing data (see Methods section)
[2] HAI: haemagglutination inhibition assay
