# Supplementary material for: Links between fecal microbiota and the response to vaccination against influenza A virus in pigs
Source: NPJ Vaccines. 2021 Jul 22;6:92. doi: 10.1038/s41541-021-00351-2 (PMC8298503; doi:10.1038/s41541-021-00351-2)
Supplement: Supplementary file 3 — Reporting Summary [file 41541_2021_351_MOESM3_ESM.pdf]

nature research

Corresponding author(s): DBPR: NPJVACCINES-01201R

Last updated by author(s): Apr 30, 2021

Reporting Summary

Nature Research wishes to improve the reproducibility of the work that we publish. This form provides structure for consistency and transparency in reporting. For further information on Nature Research policies, see our Editorial Policies and the Editorial Policy Checklist.

Statistics

For all statistical analyses, confirm that the following items are present in the figure legend, table legend, main text, or Methods section.

n/a	Confirmed	
☐	☒	The exact sample size (*n*) for each experimental group/condition, given as a discrete number and unit of measurement
☐	☒	A statement on whether measurements were taken from distinct samples or whether the same sample was measured repeatedly
☐	☒	The statistical test(s) used AND whether they are one- or two-sided<br>*Only common tests should be described solely by name; describe more complex techniques in the Methods section.*
☐	☒	A description of all covariates tested
☐	☒	A description of any assumptions or corrections, such as tests of normality and adjustment for multiple comparisons
☐	☒	A full description of the statistical parameters including central tendency (e.g. means) or other basic estimates (e.g. regression coefficient) AND variation (e.g. standard deviation) or associated estimates of uncertainty (e.g. confidence intervals)
☐	☒	For null hypothesis testing, the test statistic (e.g. *F*, *t*, *r*) with confidence intervals, effect sizes, degrees of freedom and *P* value noted<br>*Give P values as exact values whenever suitable.*
☒	☐	For Bayesian analysis, information on the choice of priors and Markov chain Monte Carlo settings
☒	☐	For hierarchical and complex designs, identification of the appropriate level for tests and full reporting of outcomes
☐	☒	Estimates of effect sizes (e.g. Cohen's *d*, Pearson's *r*), indicating how they were calculated

Our web collection on statistics for biologists contains articles on many of the points above.

Software and code

Policy information about availability of computer code

Data collection	MiSeq M04152 Illumina : MiSeq Control Software, including MiSeq Reporter (version 3.1) and Bcl2fastq2 (version 2.15.0).
Data analysis	Sequence analysis: FROGS (version3.0), Swarm (version 1.4.1), Vsearch (version 2.6.0), NCBI Blast+ (2.6.0), Mafft (version 7.310), Fasttree (version 2.1.10), fastspar, and SILVA database (version 132 from the 2017 update).<br>Statistical analysis: R (version 3.5.2), Phyloseq package (version 1.26.1), Vegan package (version 2.5.5), nlme package (version 3.1-142), metagenomeSeq package (version 1.24.1), igraph package (version 1.2.4.2), and MixOmics package (version 6.8.5).

For manuscripts utilizing custom algorithms or software that are central to the research but not yet described in published literature, software must be made available to editors and reviewers. We strongly encourage code deposition in a community repository (e.g. GitHub). See the Nature Research guidelines for submitting code & software for further information.

Data

Policy information about availability of data

All manuscripts must include a data availability statement. This statement should provide the following information, where applicable:

- Accession codes, unique identifiers, or web links for publicly available datasets
- A list of figures that have associated raw data
- A description of any restrictions on data availability

16s rRNA gene sequence data are available through the NCBI Sequence Read Archive (accession number PRJNA647267). All other data that support the findings of this study are available in the supplementary tables and from the corresponding author upon reasonable request.

Field-specific reporting

Please select the one below that is the best fit for your research. If you are not sure, read the appropriate sections before making your selection.

- [x] Life sciences
- [ ] Behavioural & social sciences
- [ ] Ecological, evolutionary & environmental sciences

For a reference copy of the document with all sections, see nature.com/documents/nr-reporting-summary-flat.pdf

Life sciences study design

All studies must disclose on these points even when the disclosure is negative.

Sample size
In total, 131 piglets were included in the study, comprising 98 vaccinated pigs (48 females and 50 males) and 33 non-vaccinated control pigs (17 females and 16 males). We did not apply a specific method to assess the statistical power of the study, but decided the sample size based on previous studies.

Data exclusions
Based on the sequencing depth and the rarefaction curves, we rarefied counts for alpha- and beta-diversity analyses at 10 000 counts per sample. After this rarefaction step, a few samples were excluded; the final dataset contained 89 animals at D28 (nD28=89), 97 animals at D49 (nD49=97), 98 animals at D63 (nD49=98), and 83 animals at D146 (nD49=83).

Replication
There were no replication for fecal DNA 16S rRNA sequencing.
For the phenotyping of the vaccine response, IAV specific IgG were measured in duplicate.

Randomization
Per litter animals were chosen in order to balance the sex ratio among the vaccinated and non vaccinated groups.

Blinding
The blinding was not relevant for our study.

Reporting for specific materials, systems and methods

We require information from authors about some types of materials, experimental systems and methods used in many studies. Here, indicate whether each material, system or method listed is relevant to your study. If you are not sure if a list item applies to your research, read the appropriate section before selecting a response.

Materials & experimental systems

n/a	Involved in the study	
[ ]	[x]	Antibodies
[x]	[ ]	Eukaryotic cell lines
[x]	[ ]	Palaeontology and archaeology
[ ]	[x]	Animals and other organisms
[x]	[ ]	Human research participants
[x]	[ ]	Clinical data
[x]	[ ]	Dual use research of concern

Methods

n/a	Involved in the study	
[x]	[ ]	ChIP-seq
[x]	[ ]	Flow cytometry
[x]	[ ]	MRI-based neuroimaging

Antibodies

Antibodies used
HRP-conjugated anti-pig IgG diluted 1/40000 (A100-104P, Bethyl Laboratories)

Validation
Describe the validation of each primary antibody for the species and application, noting any validation statements on the manufacturer's website, relevant citations, antibody profiles in online databases, or data provided in the manuscript.

Animals and other organisms

Policy information about studies involving animals; ARRIVE guidelines recommended for reporting animal research

Laboratory animals
For laboratory animals, report species, strain, sex and age OR state that the study did not involve laboratory animals.

Wild animals
Provide details on animals observed in or captured in the field; report species, sex and age where possible. Describe how animals were caught and transported and what happened to captive animals after the study (if killed, explain why and describe method; if released, say where and when) OR state that the study did not involve wild animals.

Field-collected samples
The experiment was conducted on the INRAE experimental farm at le Magneraud (GenESI, Pig Phenotyping and Innovative Breeding Facility, (doi: https://doi.org/10.15454/1.5572415481185847E12). We produced 26 litters of Large White pigs. The animals were born in five batches over a period of six months and were grown indoors. Piglets were weaned at 28 days of age on average (from 24 to 30 days of age). The animals were raised under standard conditions in pens of 20 to 30 animals during the post-weaning period

	(from around 28 to 68 days of age) and in pens of 10 to 12 animals during the growth period (from around 68 to 146 days of age). After weaning, the pigs were fed ad libitum with a commercial diet (GUSTI PRIM, NUTRICIAB®) up to 34 days of age, then with a post-weaning diet up to 68 days of age, and finally with a growth diet until slaughter. No antibiotic treatment was administered at any time during the experiment. Blood and fecal samples were individually collected.
Ethics oversight	All animal procedures were performed according to guidelines for the care and use of experimental animals (permission for animal experimentation to C. Rogel-Gaillard: A78-172; agreement for experimentation from the Centre de Recherche INRA Poitou-Charentes - Site expérimental du Magneraud: A17661; protocol approved by the French Ministry of Research with authorization ID APAFIS#4295-2016022615583351 v4 after review by ethics committee Nº 084).

Note that full information on the approval of the study protocol must also be provided in the manuscript.
